# *Pseudomonas* Exotoxin-Based Immunotoxins: Over Three Decades of Efforts on Targeting Cancer Cells With the Toxin

**DOI:** 10.3389/fonc.2021.781800

**Published:** 2021-12-16

**Authors:** Seyed Mehdi Havaei, Marc G. Aucoin, Ali Jahanian-Najafabadi

**Affiliations:** ^1^ Department of Pharmaceutical Biotechnology, School of Pharmacy and Pharmaceutical Sciences, Isfahan University of Medical Sciences, Isfahan, Iran; ^2^ Department of Chemical Engineering, Faculty of Engineering, University of Waterloo, Waterloo, ON, Canada

**Keywords:** immunotoxin, *Pseudomonas* exotoxin A, cancer, targeted therapy, bacterial toxin

## Abstract

Cancer is one of the prominent causes of death worldwide. Despite the existence of various modalities for cancer treatment, many types of cancer remain uncured or develop resistance to therapeutic strategies. Furthermore, almost all chemotherapeutics cause a range of side effects because they affect normal cells in addition to malignant cells. Therefore, the development of novel therapeutic agents that are targeted specifically toward cancer cells is indispensable. Immunotoxins (ITs) are a class of tumor cell-targeted fusion proteins consisting of both a targeting moiety and a toxic moiety. The targeting moiety is usually an antibody/antibody fragment or a ligand of the immune system that can bind an antigen or receptor that is only expressed or overexpressed by cancer cells but not normal cells. The toxic moiety is usually a protein toxin (or derivative) of animal, plant, insect, or bacterial origin. To date, three ITs have gained Food and Drug Administration (FDA) approval for human use, including denileukin diftitox (FDA approval: 1999), tagraxofusp (FDA approval: 2018), and moxetumomab pasudotox (FDA approval: 2018). All of these ITs take advantage of bacterial protein toxins. The toxic moiety of the first two ITs is a truncated form of diphtheria toxin, and the third is a derivative of *Pseudomonas* exotoxin (PE). There is a growing list of ITs using PE, or its derivatives, being evaluated preclinically or clinically. Here, we will review these ITs to highlight the advances in PE-based anticancer strategies, as well as review the targeting moieties that are used to reduce the non-specific destruction of non-cancerous cells. Although we tried to be as comprehensive as possible, we have limited our review to those ITs that have proceeded to clinical trials and are still under active clinical evaluation.

## Introduction

Cancer is the leading cause of death in developed countries and the second cause of death in developing countries (1, 2). The prevalence and mortality of cancer are rapidly growing because of aging, population growth, and predisposing behaviors such as smoking (2, 3). Over 7 million cancer-related deaths have been recorded worldwide, which is 13% of all deaths (4). Chemotherapy, radiotherapy, and surgery are the major treatment strategies for cancer (5, 6). Chemo- and radio-therapeutics target rapidly growing cancer cells; however, they are also cytotoxic to normal cells (7). Therefore, besides their success in eradicating tumor cells, conventional chemo- and radio-therapeutics result in a wide range of side effects such as alopecia, gastrointestinal symptoms, myelosuppression, and even secondary cancers (7). To reduce the side effects, various modalities have emerged that target cancer cells based on tumor-specific antigens or the use of cell-specific gene promoters (8). The aim of targeted therapy is to inhibit the proliferation of cancer cells either by delivery of growth inhibitory molecules or cytolethal agents to cancer cells or by controlled expression of cytolethal proteins *via* the use of a cancer-specific promoter (9).

In the former strategy, tumor-specific receptors, which are not, or are much less, expressed on normal cells are targeted by a monoclonal antibody, or an antibody fragment, which consequently blocks ligand–receptor interaction and intracellular signaling (10). Furthermore, the antibody/antibody fragment or the ligand can also be fused to a protein toxin to specifically kill the targeted cancer cell. Such chimeric molecules are called immunotoxins (ITs) (11). In the latter strategy, the coding sequence of a toxic protein is cloned under the control of a tumor-specific promoter and delivered to cancer cells (12). Tumor-specific promoters are derived from genes that are specifically and ectopically overexpressed in cancer cells (13). Although normal cells might uptake the expression cassette (depending on the gene delivery vehicle), transcription can be controlled by the cell type, resulting in expression in only cancer cells (14).

Targeted cancer therapy is an extremely broad area, and novel strategies are continually emerging. This review aims to highlight advances in applications of *Pseudomonas* exotoxin A (PE) protein and its derivatives for the production of ITs and their use in targeted cancer therapy. It is worth mentioning that the coding sequence of this bacterial toxin has been also used in many studies for cancer cell-specific gene therapy as summarized in **Table 1**. However, here, we only focus on various PE-derived ITs, one of which has been recently approved for clinical application.

**Table 1:** A summary of studies on gene therapy applications of *Pseudomonas* exotoxin A or its derivatives usually encoded under the control of a tumor-specific promoter for targeted killing of corresponding cancer cells.

Gene therapy modality	Applied *Pseudomonas* exotoxin A (PE) fragment	Targeted cancer	Extent of the study	Reference(s)
pULI100 DNA (adenov. mediated)	PE	Breast cancer	*In vitro*	(15)
pCMV-e23sFv-PE40 (transduced lymphocyte)	PE40	Breast cancer	*In vitro* and *in vivo*	(16)
pWF-47-TEG (plasmid bound to TGF-alpha)	PE	various tumors	*In vitro*	(17)
Retro-1.3MBP-pe-toxin (under thyroid hormone promoter)	PE	Brain tumor	*In vitro* and *in vivo*	(18)
pPETOPN	The receptor-binding and membrane translocation moiety is PE	various tumors	*In vitro*	(19)
sigVEGFPE/pcDNA.3	PE	Myeloid tumors	*In vitro* and *in vivo*	(20)
pCMV-ETA-EGFP	ETA	cancers	*In vitro*	(21)
Ad.mhIL-4TRE.mhIL-13-PE	PE	Glioma	*In vitro* and *in vivo*	(22)
pVEGF165PE38-IRES2-EGFP	PE38	Glioma	*In vivo*	(23)
pGene/V5-His-ETA	ETA	Head and neck	*In vitro*	(24)
PM/pG-CM-PE, PM/pG-CM-bF-PE, PM/pG-CM-CX-PE, PM/pF-CX-bF-PE	PE	Breast cancer	*In vitro*	(25)
pSERPINB3-PE38KDEL	PE38KDEL	Oral squamous cell carcinoma	*In vitro*	(12)

## Immunotoxins

ITs were first defined as engineered proteins consisting of an antibody or antibody fragment, or a ligand of the immune system, such as a growth factor or cytokine, as the targeting moiety, fused to a cytolethal protein. However, recent studies have used other cancer-specific targeting molecules including natural or synthetic cell-penetrating peptides (26, 27) as well as natural and mutated antimicrobial peptides (28, 29). The toxic moiety has not deviated as much and has consisted of cytolethal proteins from plants, animals, fungi, or bacteria (30). Among the bacterial toxins, diphtheria toxin (9, 31, 32), Shiga toxin (33), *Vibrio cholerae* toxin (34), and PE (35, 36) have been used for construction of ITs. PE is one of the most potent bacterial toxins and is the most toxic virulence factor of the pathogenic bacterium *Pseudomonas aeruginosa*.

## *Pseudomonas* Exotoxin A

PE is an NAD^+^-diphthamide-ADP-ribosyl transferase (EC 2.4.2.36), which falls within the family of mono-ADP-ribosyl transferases (37). PE ADP-ribosylates eukaryotic elongation factor-2 (eEF-2) on the ribosomes when it reaches the cytosol of eukaryotic cells (38). eEF-2 is a crucial factor involved in protein biosynthesis and promotes GTP-dependent translocation of mRNA from the ribosomal A-site to the P-site (39). ADP-ribosylation results in the inactivation of eEF-2 and subsequent termination of protein biosynthesis within the affected cell. Consequently, extrinsic and intrinsic apoptosis pathways are activated, which results in cell death (40–42).

**Figure 1:**
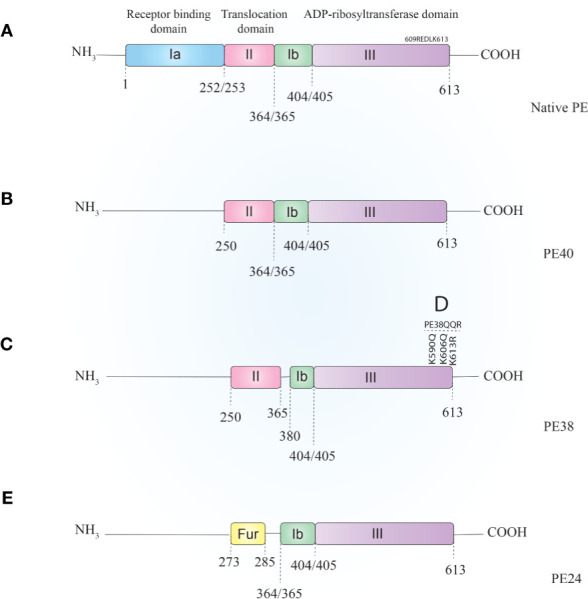
Schematic representation of *Pseudomonas* exotoxin A **(A)** and its most applicable derivatives. In order to reduce PE–ITs, non-specific toxicities, immunogenicity, and size, various PE derivatives have been evaluated, most of which are PE40 **(B)**, PE38 **(C)**, PE38QQR **(D)**, and PE24 **(E)**. PE, *Pseudomonas* exotoxin A; IT, immunotoxin.

PE is a 638-amino-acid protein that belongs to the AB toxins family (**Figure 1A**), where the A domain harbors enzymatic activity and the B domain acts as a cell-binding moiety (43, 44). The first 25 amino acids form a highly hydrophobic signal sequence that is removed during secretion (5). The remaining 613 amino acids make up three domains. Domain Ia (aa 1–252) is a receptor-binding domain and helps PE to recognize and attach to target cells. Domain II (aa 253–364) facilitates translocation of PE across the cell membrane and contains a furin cleavable motif (aa 274–280, RHRQPR^G). The last four residues (amino acids 400–404) of domain Ib (aa 365–404) along with domain III (405–613 aa) make up the catalytic part of the toxin (45). Upon secretion, the terminal amino acid residue of PE (lysine 613) is thought to be cleaved by a host plasma carboxypeptidase, which converts the REDLK (609–613) motif into REDL (609–612) (as reviewed by 37). PE interacts with the low-density lipoprotein receptor-related protein 1 (LRP-1) (aka CD91 or the α2-macroglobulin receptor) *via* its Ia domain and is subsequently internalized through receptor-mediated endocytosis. In the early endosome, PE dissociates from the receptor and undergoes a conformational change due to the acidic environment. This makes the furin-cleavable motif accessible, which allows furin to cleave PE into two fragments of about 28 kDa (aa1–279) and 37 kDa (aa280–613) (45). The smaller fragment consists of domain Ia and parts of domain II. The larger fragment contains parts of domain II, domains Ib, and domain III and has enzymatic activity. The 37-kDa fragment exploits a pathway from the late endosome to the trans-Golgi network (TGN) and from there to the ER *via* retrograde pathway following interaction of its C-terminal REDL (aa 609–612) motif with the KDEL receptors on the TGN (46, 47).

### *Pseudomonas* Exotoxin A-Derived Immunotoxins

**Figure 2:**
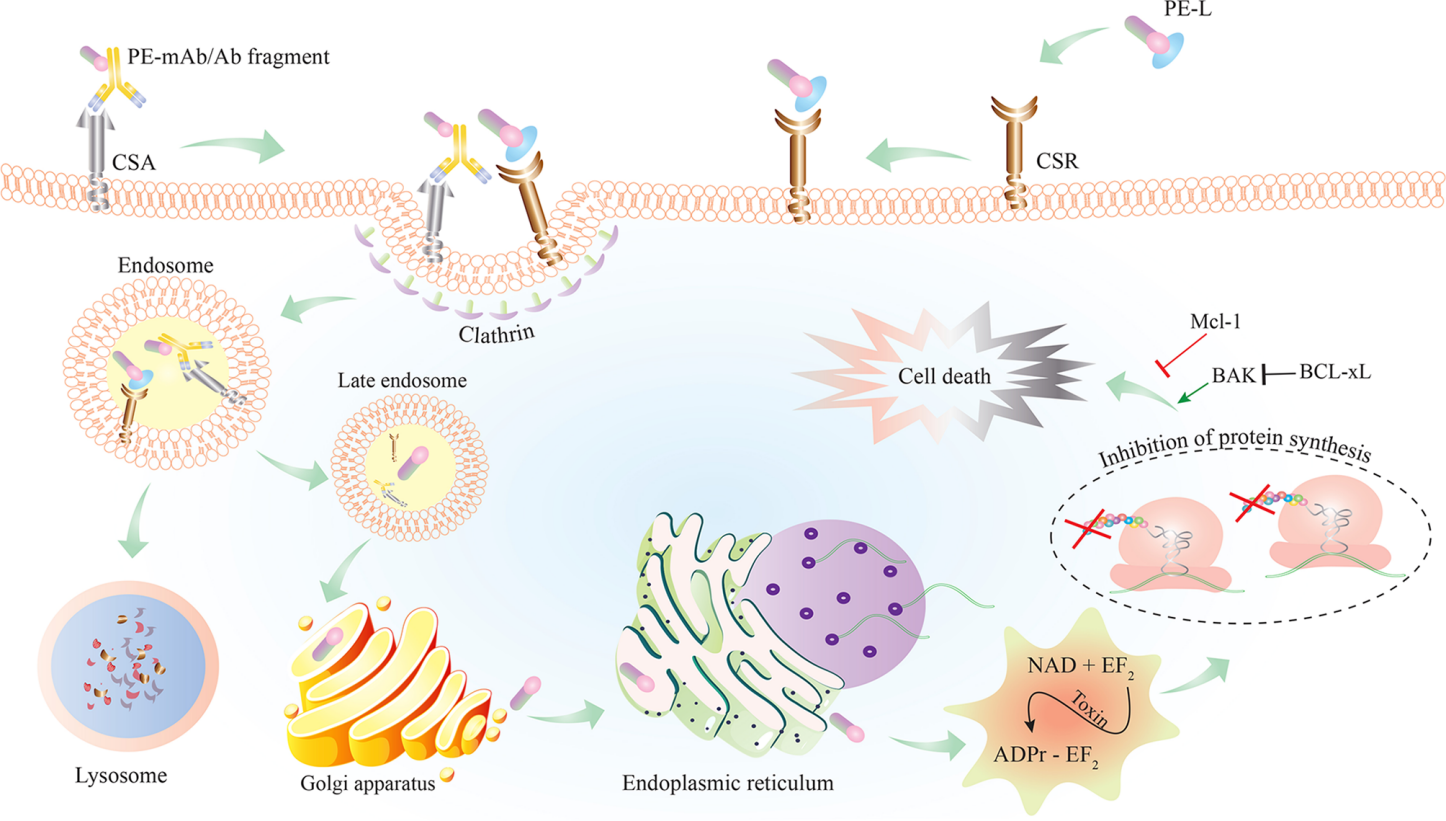
Schematic representation of PE–IT interaction with the cancer-specific antigen (CSA) or cancer-specific receptor (CSR) targeted on cancer cells and the subsequent intracellular events resulting in cell death. PE-L, *Pseudomonas* exotoxin A (PE) fused to a cancer-specific ligand; Ab, antibody; IT, immunotoxin.

To date, various ITs have been constructed by chemical conjugation or recombinant fusion of full or truncated PEs to target moieties (**Figure 2**). Chemically conjugated ITs lacked stability (due to vulnerable disulfide or thioether bonds used for chemical conjugation) and specificity (due to having the native binding domain of PE in addition to having the conjugated targeting moiety) (48, 49). Attempts have been made to produce more stable and specific PE–ITs *via* recombinant protein production.

PE40 and PE38 (**Figures 1B, C**, respectively) are the two most-used truncated forms of PE, constructed by the removal of the Ia domain, or the Ia and part of the Ib domains (aa 365–380), respectively. Moreover, to enhance ER localization of the PE-derived ITs, the C-terminus REDL motif of PE has been mutated to match the KDEL receptors on TGNs (PE-KDEL) (46, 50). Studies involving “PE-derived immunotoxins” retrieved by searching scientific databases have been collated in **Supplementary Table 1**. PE-derived ITs have been evaluated *in vitro*, *in vivo*, and/or in clinical studies. Although the list is comprehensive, this review has been limited to those therapeutics that have advanced to clinical evaluations and that are currently under active clinical trial or clinical application. In **Table 2**, PE-derived ITs that proceeded to phase I clinical trial are described. However, only a few of them have been safe enough to advance to subsequent phases.

**Table 2:** PE-derived immunotoxins advanced to various phases of clinical evaluations or clinical application.

Immunotoxin	Toxin fragment	Targeting moiety	Target disease	Extent of study	Reference
Moxetumomab	PE38	Anti-CD22	Hairy cell leukemia	FDA-approved	(36)
LMB-100	PE38	Anti-mesothelin	Pancreatic adenocarcinoma	Clinical trial phases 1 and 2	(51)
IL-13-PE38QQR	PE38QQr	IL-13	Glioblastoma multiforme	Clinical trial phase 3	(52)
CD4-PE40	PE40	CD4	HIV	Clinical trial phase 3	(53)
LMB-2	PE38	Anti-IL2 (anti-TAC)	Hematological malignancies	Clinical trial phase 2	(54)
Erb-38	PE38	Fv portion of MAb e23	Breast cancer and esophageal cancer	Clinical trial phase 1	(55)
D2C7-IT	PE38KDEL	EGFR	Glioblastoma	Clinical trial phase 1	(56)
LMB-1	PE38	B3	Solid tumors	Clinical trial phase 1	(57)
NBI-3001	PE38KDEL	IL4	Solid tumors	Clinical trial phase 2	(58)
SGN-10	PE40	BR96	Advanced solid tumor	Clinical trial phase 1	(59)
VB4-845 (oportuzumab monatox or Vicinium)	PE	Anti-EpCAM	Squamous cell carcinoma of the head and neck	Clinical trial phase 3	(60)
OVB3-PE	PE	OVB3	Ovarian cancer	Clinical trial phase 1	(61)

PE, Pseudomonas exotoxin A; FDA, United States Food and Drug Administration; EGFR, epidermal growth factor receptor.

In the next sections, we review those PE–ITs that are currently under active clinical trials or approved for clinical application. In each case, first, the targeted receptor and its significance in the corresponding malignancy or disease will be addressed, and then, the IT(s) will be discussed.

### Targeting CD22

CD22 is an inhibitory co-receptor of the B-cell receptor that is expressed on the surface of normal B lymphocytes and also many malignant B cells including chronic B-lymphocytic cells (B-CLL), B-lymphoma cells such as Burkitt’s lymphoma, and hairy cell leukemia (HCL). However, because CD22 is not expressed on stem cells, it has been considered for targeting the malignancies (62).

RFB4 is a monoclonal antibody against CD22, which was primarily used for the production of chemically conjugated PE–ITs (63). However, in order to enhance tumor penetration as well reduce non-specific cytotoxicity of the ITs, the chemically conjugated ITs were replaced by a recombinant IT (RFB4(dsFv)PE38) composed of disulfide stabilized variable fragments (dsFv) of the RFB4 antibody fused to PE38. The recombinant IT showed IC_50_ values of approximately 2 ng/ml on four Burkitt’s lymphoma cell lines, while being not toxic to CD22-negative cell lines (IC_50_ > 1,000 ng/ml) (64). Next, *in vivo* studies in nude mouse xenograft of human Burkitt’s lymphoma resulted in 80% and 100% tumor regression following administration of 200 or 275 µg/kg of the IT every other day for three doses. The cumulative MTD of the IT was 3.7 mg/kg for continuous infusion and 1.2 mg/kg for intermittent bolus dosing. In addition, i.v. infusion of three doses of 275 or 350 μg/kg every other day of RFB4(dsFv)PE38 resulted in complete remission (CR) in all animals. In the case of continuous infusion, 100 or 200 μg/kg/day resulted in CR (65).

Safety and specificity of the IT were also studied in cynomolgus monkeys that also express CD22, similar to humans. Three doses of 100 or 500 μg/kg injected every other day were well tolerated and showed mild laboratory abnormalities. The tolerated dose of 500 μg/kg was much higher than the dose needed to obtain CR in the mice xenografts (i.e., 275 μg/kg) (65).

The level of cytotoxicity of the IT was also confirmed on cell samples obtained from 28 patients with CD22-positive leukemias. Moreover, the sensitivity of CD22-positive tumor cells was correlated with CD22 expression on the cells’ surface (66).

RFB4(dsFv)-PE38 proceeded to clinical evaluations under the name of BL22 (aka CAT-3888). In a dose-escalation trial (Clinicaltrials.gov Identifier (CTI): NCT00021983) on 16 patients with chemotherapy-resistant HCL, 69% (11 patients) underwent CR and 12% (two patients) had partial remission (PR) following administration of 3–50 µg/kg of the IT, every other day for three times in a cycle, and within a range of 1 to 15 cycles in different patients. The three non-responsive patients had neutralizing antibodies before therapy or received the least amount of the therapeutic (up to 6 µg/kg/dose) (67). Moreover, only one case of dose-limiting cytokine release syndrome was reported. Therefore, the IT was further evaluated in a phase I trial (CTI: NCT00126646) on 46 CD22+ patients (four non-Hodgkin’s lymphoma (NHL), 11 chronic lymphocytic leukemia (CLL), and 31 HCL). The effective and tolerable dose of BL22 consisted of one cycle (three doses) of 40 μg/kg of the IT every other day. However, among the patients, only HCL patients responded with 19 (61%) CRs and six (19%) PRs (81% of overall response), likely because of the higher number of CD22 on the surface of HCL cells (5,000 to 70,000) compared with CLL (350–1,000) and NHL cells (unknown) (68).

BL22 proceeded to a phase II clinical trial (CTI: NCT00074048) consisting of 36 chemoresistant HCL patients. The patients received a single round of treatment consisting of 40 μg/kg of the IT every other day over 5 days (total of three doses). This resulted in nine cases (25%) with CR, one of which relapsed. After the re-treatment of 20 patients (56%), the total CRs increased to 17 CR (47%). Therefore, the high response rate seen in HCL patients during phase I testing was also confirmed by the phase II trial. In addition, for both phase I and phase II trials, the average dose per cycle was similar, ranging from 29 to 33 μg/kg three times in a cycle. In phase II, hemolytic uremic syndrome (HUS) occurred less than in phase I, and major toxicities were hypoalbuminemia, aspartate aminotransferase/alanine aminotransferase (AST/ALT) elevation, edema, myalgia, proteinuria, fatigue, nausea, and fever (69).

BL22 was also evaluated in patients with B-cell acute lymphoblastic leukemia (ALL) that relapsed after chemotherapy. A phase I dose-escalation trial (CTI: NCT00077493) on 23 pediatric patients who received 10–40 µg/kg of BL22 every other day for three or six doses repeated every 21 or 28 days revealed a significant reduction in peripheral blast counts, recovery of normal blood counts, or decreased blast infiltration of bone marrow and extramedullary sites in 16 subjects (70%). However, although the treatment was associated with an acceptable safety profile, and adverse events were rapidly reversible, higher doses might have been required to achieve maximal benefit (70).

As mentioned before, the very low efficiency of BL22 in CLL patients is likely due to the small quantity of CD22 on the surface of CLL cells. In an attempt to increase the cytotoxicity of BL22, a greater affinity toward CD22 was sought. Phage display was used to screen various mutations in the heavy chain (VH) complementarity-​determining regions 3 (CDR3) of RFB4(dsFv). Among five selected mutants, a variant where the CDR3 Ser-Ser-Tyr (SSY) residues were replaced by Thr-His-Trp (THW) showed increased affinity (~14-fold) toward the target molecule compared with the parent protein. When the THW mutant was fused to PE38, the IC_50_ values on fresh leukemic cells from CLL and HCL patients decreased to 22–29 ng/ml compared with an IC_50_ > 1,000 ng/ml for BL22 on the same cells. This 50-fold difference in IC_50_ (71) prompted additional *in vitro* and *in vivo* evaluations of RFB4(dsFv) THW mutant-PE38, now also known as HA22 or CAT-8015 (72). HA22 cytotoxicity was evaluated on Burkitt’s lymphoma and CLL cell lines. An overall IC_50_ of 0.3–8.6 ng/ml, with only 10% cell survival following treatment with ≥50 ng/ml of HA22 was determined. Safety studies were performed in cynomolgus monkeys receiving similar doses of either HA22 or BL22, and no differences have been reported between the two ITs. This suggested that the increased affinity of CAT-8015 for CD22 was not associated with additional or exacerbated side effects. *In vivo* antitumor activity was evaluated in female athymic NCr nude mice model of human Burkitt’s lymphoma. Administration of three doses of 12.5 μg/kg of HA22 injected every other day resulted in a long-lasting cytostatic response. Moreover, when the dose increased to 150 μg/kg with the same dosing paradigm, a long-term inhibition in the growth of Burkitt’s lymphoma was achieved. Therefore, the non-clinical data suggested that a therapeutic window for HA22 in humans could be achieved (73).

HA22, under the name of moxetumomab pasudotox (aka Moxe), proceeded to a phase I dose-escalation trial (CTI: NCT00462189) consisting of 28 patients with chemotherapy-resistant HCL. The patients received cycles of three doses of 5 to 50 µg/kg every other day, for one to 16 cycles (median: four cycles). It must be noted that 50 µg/kg (administered in 12 of the patients) is one dose level higher than the MTD of BL22. No dose-limiting toxicity (DLT) was reported, and only two cases of reversible HUS were observed. Hypoalbuminemia (in 54% of total treatment cycles) and elevated aminotransferases (38% for both ALT and AST) were the most commonly observed drug-related adverse effects. Hence, HA22 showed an acceptable safety profile in patients at the given doses. Of the patients, 86% responded to the treatment, with 46% having CR, even with doses as low as 10 µg/kg for a single cycle, and 39% having PR. The median duration of response was 29 months, and only one patient relapsed within 1 year of treatment. Moreover, seven patients in CR were negative for minimal residual disease (MRD) (74). The phase I trial was further expanded to a long-term follow-up (CTI: NCT00586924) by the inclusion of 21 patients receiving 50 µg/kg of the IT every other day for three doses in 4-week cycles. Of the 21-patient extension, 91% responded to treatment, with 71% having CR, improving the overall study to an 88% overall response rate and 64% having CR. Among the 21 patients with CR, 11 (55%) cases were negative for MRD. The median CR duration was 82.7 months in MRD-negative CRs (75). Moxe was further evaluated in a phase III pivotal, multicenter, single-arm, open-label study at 32 centers in 14 countries (CTI: NCT01829711). A dose of 40 μg/kg was administered by intravenous (i.v.) infusion over 30 min on days 1, 3, and 5 of 28-day cycles for a maximum of six cycles, or documentation of MRD-negative CR, disease progression, initiation of alternate therapy, or unacceptable toxicity (75). The reason for the dose reduction (50 to 40 μg/kg) was due to the improvement of the Moxe production process, which resulted in higher IT activity (76). A primary report from this pivotal study at a median follow-up of 16.7 months (range: 2.1–48.8 months) showed enrolment of eighty patients, among which 33 (41%) achieved CR. Moreover, 24 (30%) of the CRs were durable (CR with hematologic remission >180 days), and 27 (33%) of the CRs were negative for MRD by immunohistochemistry. The median duration of MRD-positive CR was 5.9 months. The most common treatment-related adverse events leading to permanent discontinuation (n = 8) were HUS, capillary leak syndrome (CLS), and increased blood creatinine. All HUS and CLS events were reversible (77). In Sep 2018, Moxe (Lumoxiti™) was approved by the US FDA for the treatment of adult patients with relapsed or refractory HCL who received at least two prior systemic therapies, including treatment with a purine nucleoside analog (36). Lumoxiti™ obtained European Medicines Agency (EMA) approval for the treatment of HCL patients with the same conditions in Dec 2020 (78). The final report on the phase III study was recently released, which covered a median follow-up period of 24.6 months (range: 1.2–71.7 months). According to the report, the safety concerns were similar to the previous report, but the efficacy data were better, with the rate of durable CR increasing to 36% (vs. 30% in the primary report). In addition, a durable CR rate with HR ≥ 360 days was reported in 33% of the patients (79).

Like BL22, Moxe was also evaluated in pediatric B-lineage ALL malignancies, where blasts from the patients were shown to be sensitive to Moxe (70), and the data provided enough rationale for the IT to be clinically evaluated in children with resistant ALL (80). Therefore, the safety and efficacy of Moxe were evaluated in a phase I multicenter dose-escalation trial (CTI: NCT00659425) followed by a phase II study (CTI: NCT02227108) in children, adolescents, and young adults (ages 1–25 years) with ALL or NHL. However, due to the drug-related adverse effects and unacceptable clinical activity of the IT, the study was terminated prior to a planned interim analysis (81). Therefore, although Moxe was promising for the treatment of relapsed HCL patients and even got approval for the treatment of the disease, it was not deemed to be safe and effective enough for use in pediatric B-lineage ALL. Further investigations are warranted for the latter.

### Targeting Interleukin 13 Receptor

IL-13 is predominantly a TH2-derived immunoregulatory cytokine. Since IL-13 receptors are significantly overexpressed on glioma cells (more than 30,000 receptors/cell), a PE-derived IT called IL-13-PE38QQR (**Figure 1D**) was constructed (82). The PE38 fragment in this IT harbored three mutations: C-terminal lysines at positions 590, 606, and 613 were mutated to glutamine, glutamine, and arginine, respectively. This modification of PE38 enhanced the toxicity of the protein and increased its expression in *Escherichia coli* during production (83). The cytotoxicity of the IT was assessed in nine glioma cell lines with IC_50_ values ranging between <0.1 and >300 ng/ml. Furthermore, the cytotoxicity was blocked in the presence of IL-13, which confirmed the specificity of the IT (84).

One of the limitations for targeting brain tumors is the delivery of therapeutic agents through the blood–brain barrier (BBB) (85). IL-13-PE38QQR has suffered this problem due to the size of the molecule. To overcome this limitation, convection-enhanced delivery (CED) has been used. CED facilitates the distribution of macromolecules into brain tissue by positive-pressure micro-infusion over a period of hours to days (86). In a human glioma xenograft model that received 100 µg/kg of IL-13-PE38QQR *via* CED, all six animals showed CR without acute histopathologic cytotoxicity (87). The safety of IL-13-PE38QQR was assessed in rats receiving 24-h infusion (200 µl totally) of a 10 μg/ml solution of the IT. All mice (n = 6) tolerated the infusion without any neurological changes (88).

The safety and efficacy of IL-13-PE38QQR were further evaluated clinically under the name cintredekin besudotox (CB). In a phase I trial (CTI: NCT00024570) consisting of 51 patients with recurrent malignant gliomas who received 0.25, 0.5, or 1 µg/ml of the IT *via* CED over 5–6 days of infusion, the 1 µg/ml dose caused DLT. The doses of 0.25 and 0.5 µg/ml were very well tolerated, and no patients displayed any toxicity. The adverse events were neurologic or psychiatric including headache, sensory disturbance, aphasia, asthenia, and convulsion (89). In a phase III clinical trial (CTI: NCT00076986), the efficacy of administration of CB *via* CED was compared with an approved treatment (Gliadel wafers containing 7.7 mg of carmustine) in adults with recurrent glioblastoma multiforme (GBM). Although the median survival duration in patients who received CB was higher than in patients who received standard treatment (9.1 *vs.* 8.8 months), the incidence of severe adverse effects, especially thromboembolic complications, resulting in death or termination of the treatment was higher in cases treated with CB. Poor results of CB could also be attributed to the need for exact placement of catheters, which were incorrect in up to 49% of the cases (52, 90). A later animal study comparing CED with bolus stereotactic administration resulted in higher volume distribution in the tumor tissue that lasted for a longer period of time (91); however, improper positioning of the catheter, the catheter configuration, the infusion rate, and the infusion volume could have all contributed to inadequate concentration of CB in tumors and consequently lower than expected efficiencies. Moreover, the GBM patients were not assessed for the density of IL-13 receptor on their tumor cells (52). Variation in the expression level of the receptor among the patients and on the tumors (as shown before (92, 93) could be another reason for the lackluster results.

In addition to GBM, a dose-escalation phase I trial (CTI: NCT00088061) to evaluate the safety and tissue distribution of the IL13-PE38QQR administered *via* CED in five children with diffuse intrinsic pontine glioma (DIPG) has been reported recently. However, despite interim prevention of DIPG progression in two of the patients, all patients showed disease progression 3 months after initial infusion and died (94). There has been no further report on any aspects of IL13-PE38QQR in DIPG patients.

### Targeting Mesothelin

Mesothelin is a cell surface antigen that is highly expressed on pancreatic, colon, lung, and ovarian solid tumors as well as in mesothelioma and cholangiocarcinoma (95). The physiological role of mesothelin is not known, but it might be involved in tumorigenesis and metastasis (96–98). The antigen was first detected in a search for monoclonal antibodies interacting with ovarian cancer cells but not normal human tissues, *via* immunization of mice with a human ovarian cancer cell line, OVCAR3 (99). Later, it was shown that mesothelial cells are the only normal cells that express mesothelin, albeit at a much lower level than on malignant cells (100).

To target and destroy these tumors, K1, a monoclonal antibody against mesothelin, (99, 101) was conjugated to PE38QQR (K1-LysPE38QQR). *In vitro*, the IC_50_ values of 3–6 ng/ml were obtained for A431-K5 cells (a cell line engineered to express mesothelin). *In vivo*, complete tumor regression was observed in 50% of A431-K5 xenograft nude mice that received three doses of 0.75 mg/kg of the IT (102). Further studies aimed at the production of K1-based ITs by recombinant DNA technology. In this regard, the single-chain variable fragment (scFv) of the K1 antibody was fused to PE38. The scFv-PE38 showed reasonable cytotoxicity on A431-K5 cells with an IC_50_ of 0.6 ng/ml, whereas on various mesothelin-negative cells, IC_50_ ranged between 450 and 1,000 ng/ml. Unfortunately, the IT was very unstable with a half-life of 8 h at 37°C (103). Therefore, a series of attempts were made to produce an anti-mesothelin scFv with enhanced stability, binding characteristics (k_D_), and cytotoxicity (when fused to PE38) (103–105). Finally, an improved scFv with high stability (up to 40 h at 37°C) and high affinity (k_d_ of 11 nM) was obtained and designated as SS scFv. In addition, when fused to PE38, the SS scFV-PE38 IT had an IC_50_ of 0.5 ng/ml on A431-K5 cells and 6–16 ng/ml on mesothelin-positive cancer cells. The IC_50_ ranged from 450 to over 1,000 ng/ml on mesothelin-negative cancer cells. An *in vivo* study in A431-K5 xenograft nude mice, i.v. injected with three doses of 2.6 or 4.3 µg of the IT every other day, showed complete tumor regression (103). Next, random mutations were introduced to hotspots in the complementarity-determining region 3 (CDR3) of the light chain of SS scFv to enhance its affinity. Finally, a variant named SS1 showed much higher affinity (15 times) and when fused with PE38 (SS1 scFv-PE38) showed an increase in *in vitro* cytotoxicity (13 times) (106). In another study, a disulfide bond stabilized (ds) bivalent SS Fv (SS (dsFv)_2_) was developed in which the V_L_ and V_H_ of each Fv were joined *via* a disulfide bond. The two Fvs were fused to PE by a (Gly_4_-Ser)_3_ linker. The SS (dsFv)_2_-PE38 IT had an enhanced half-life (47 vs. 27 min), affinity (40 times more), and *in vitro* cytotoxicity (10 times higher) when compared with SS dsFv-PE38. However, no significant difference in activity was reported between the two ITs *in vivo* (107). Combining advances, SS1(dsFv)-PE38 was constructed and evaluated on the primary culture of tumor cells obtained from patients with ovarian and cervical cancers. The IC_50_ of SS1(dsFv)-PE38 was 1–10 ng/ml for mesothelin-positive cells, while for negative cells, the IC_50_ was greater than 1,000 ng/ml (108). Moreover, tumor cells obtained from ascites of patients with peritoneal mesothelioma were killed by the IT with IC_50_ values ranging between 0.08 and 3.9 ng/ml (109).

*In vivo* evaluation of SS1(dsFv)-PE38 in nude mice xenografted with non-small cell lung cancer (NSCLC) cells resulted in almost complete tumor regression and significantly prevented metastasis (110). Safety and toxicology studies of the IT were performed in cynomolgus monkeys receiving three doses of 250 or 1,000 µg/kg every other day. Decreased appetite and physical activity were the only side effects observed with the 1,000 µg/kg dose. Microscopic evaluation of serosal membranes revealed micro-inflammatory lesions, predicting pleuritic, and/or pericarditis as possible DLTs (111).

SS1(dsFv)-PE38 was termed SS1P and evaluated in a phase I dose-escalation clinical trial (CTI: NCT00006981) on patients with advanced mesothelioma, ovarian, and pancreatic cancers. The patients in different cohorts received three doses of 8–60 µg/kg every other day for either six cycles (8–25 µg/kg doses) or three cycles (25–60 µg/kg doses). However, despite the reasonable safety of the IT, it showed limited efficacy due to anti-IT antibody formation in almost 88% of the patients (111). Considering the high concentration of anti-SS1P antibodies (defined as 75% or more neutralization of SS1P activity *in vitro*) developed in most of the patients, and the limited efficiency of the IT, later studies used different strategies including 1) administration of the SS1P by continuous infusion instead of bolus injection; 2) administration of SS1P in combination with other chemotherapeutics to enhance tumor penetration and lower the required IT dose; and 3) re-engineering SS1P to remove its B- and T-cell epitopes.

The first strategy was used in a phase I clinical trial (CTI: NCT00024674) consisting of 24 patients with various mesothelioma and ovarian and pancreatic carcinoma. Patients received 4 to 25 μg/kg/day of SS1P by continuous infusion over 10 days. This strategy did lead to slightly lower neutralizing antibodies following the infusion, and the total amount of SS1P administered to the patients was higher than that of the bolus injection. However, the response in patients did not differ significantly as compared with the previous trial (112).

As the second strategy, the combination of SS1P with other chemotherapeutics was targeted. The use of chemotherapeutics has been shown to increase the uptake of IT, presumably through endothelial damage (113), making this approach a potential strategy for SS1P. In an athymic nude mice study, where the mice were xenografted with A431-K5 cells, the use of a single dose of Taxol (50, 20, or 10 mg/kg), cisplatin (5 mg/kg), or cyclophosphamide (15 mg/kg) 24 h before administration of the first dose of SS1P was investigated. As expected, results showed an increased potency and enhanced tumor regression when SS1P administration was combined with a single dose of any of the chemotherapeutics. Interestingly, complete tumor regression for at least 40 days was reported when SS1P was administered after a single dose of 20 or 50 mg/kg of Taxol. Further analysis of the results indicated a synergistic effect for the combinations. However, *in vitro* assessment of the combinations on A431-K5 cell line revealed no enhanced cytotoxic effects when compared with the agent alone, indicating that the observed synergistic effects must not be related to direct effects of the agents on the cells (114). A subsequent mechanistic study showed that the promoting effect of Taxol on SS1P efficiency was not related to endothelial damage. In Taxol-sensitive tumors, mesothelin shedding into the tumor environment and in blood was significantly reduced (~10 times) over 5 days after a single injection of 20 mg/kg of Taxol. Hence, SS1P was not antagonized by shed mesothelin and could readily bind membrane mesothelin and kill tumor cells (115). A phase I dose-escalation clinical trial (CTI: NCT01445392) has also been reported for SS1P combination therapy, with standard doses of pemetrexed and cisplatin in patients with confirmed malignant pleural mesothelioma. However, although pretreatment of the patients with the chemotherapeutics followed by administration of different doses (25–55 µg/kg) of SS1P caused some partial responses, the production of neutralizing antibodies by the patients’ immune system hampered further administration of SS1P (116). Therefore, the third strategy, i.e., de-immunizing SS1P, was intensely pursued.

De-immunization of SS1P was expected to be promising since previous studies on de-immunization of PE (117, 118) or using lymphocyte depleting regimen (119) showed reduced production of anti-SS1P antibodies and consequently increased SS1P serum concentration. These findings were confirmed by a subsequent pilot clinical trial in patients with refractory malignant mesothelioma, in which the patients received the immune-depleting regimen before each cycle of SS1P administration to reduce their B and T lymphocytes. Lower serum anti-SS1P antibody, higher serum SS1P concentrations, and prolonged PRs were found in the pilot study, supporting the notion that reducing immune responses toward the SS1P could allow repeated administration of the IT and enhance overall responses (120). Among various mutants of SS1P (99, 105, 107, 111, 112, 114–119, 121–123), RG7787 is one of the most widely studied forms, which also proceeded to clinical evaluation.

RG7787 (also known as LMB-100) is a mutant of SS1P developed by 1) introducing seven point mutations to the catalytic domain of PE (domain III), which removed its human B-cell epitopes; 2) removing the majority of domain II of PE, which removed protease sites as well as additional B-cell epitopes; and 3) replacing the murine dsFv with a humanized anti-mesothelin Fab fragment (124). The first two modifications resulted in a new generation of PE–IT based on PE24 (**Figure 1E**), which, in addition to much lower immunogenicity, was resistant to lysosomal degradation and resulted in a substantial reduction in off-target toxicity (125).

The cytotoxicity and antitumor effects of LMB-100 were verified on different cancer types including breast (126), gastric (126), lung (127), ovarian (128), and colorectal (129) cancers, as well as pancreatic ductal adenocarcinoma (130–133). Almost all cell lines corresponding to these malignancies were affected by LMB-100 at concentrations in the picomolar range (as low as 6.8 pmol/lit) (130–133). Because *in vivo* evaluation in rodents and cynomolgus monkeys revealed a 5- to 10-fold higher MTD for LMB-100 when compared with the SS1P (124, 126), LMB-100 could be administered at higher doses (2.5 vs. 0.4 mg/kg/dose for SS1P). However, although the treatment of xenograft models with LMB-100 alone resulted in some PRs and tumor regression, no cases of CR were reported (124, 126). Therefore, LMB-100 was evaluated in combination with other chemotherapeutics, including taxanes (paclitaxel and nano-albumin bound (Nab)-paclitaxel) (126–128, 130, 134), platinum-based agents (cisplatin and oxaliplatin) (128, 129), actinomycin D (129, 135), and gemcitabine (134). Co-administration of LMB-100 with the taxanes enhanced the antitumor activity in a synergistic manner and even resulted in four CRs in gastric cancer (received paclitaxel) (126), as well as pancreatic cancer (received nab-paclitaxel) (134) xenografts. Likewise, actinomycin D co-administration synergistically potentiated the antitumor activity of LMB-100 in pancreatic (135) and colorectal (129) carcinoma xenografts and resulted in some CRs. Cisplatin co-administration in an ovarian xenograft model also enhanced antitumor activity of LMB-100, though in an additive manner and without any CRs (128). The combination of gemcitabine only slightly improved LMB-100 activity in pancreatic cancer xenografts (134), and oxaliplatin showed no enhancement in antitumor activity in colorectal xenografts (129). Taken together, enhancement of LMB-100 efficacy is dependent not only on the selected chemotherapeutics but also on the type of cancer itself. None of the above studies could clearly define the mechanisms behind the observed synergistic effects, though further mechanistic and pharmacokinetic studies are underway (136, 137).

LMB-100 monotherapy was also shown to be inefficient in a phase I dose-escalation clinical trial (CTI: NCT02798536) due to the development of anti-LMB-100 antibodies, as well as the lack of durable responses or CRs in patients with different mesothelin-positive cancers (138). Further clinical evaluation of LMB-100 alone or in combination with nab-paclitaxel in a phase I/II clinical study (CTI: NCT02810418) in pancreatic cancer patients was also underwhelming (139). The study included two arms based on the infusion rate of LMB-100. In arm A (A1 and A2) of the study, patients were infused over 30 min (short infusion), while in arm B (B1 and B2), patients were infused over 24 or 48 h (long infusion). Patients in A1 (dose-escalation phase I trial), A2 (phase II trial), and B2 received nab-paclitaxel (125 mg/m^2^) in addition to the IT. Despite the beneficial antitumor activity of the combination therapy, the amplification of toxic side effects by nab-paclitaxel, especially the CLS-related side effects, such as severe myalgia and cardiac toxicity, prevented its further use (51). A recent abstract presented at the annual meeting of the American Society of Clinical Oncology (2020) reported results corresponding to the B1 and B2 arms of the study. Despite being well tolerated by the patients, higher titers of anti-IT antibodies were reported compared with the short infusion (the A arm of the study). Clinically, the combination of LMB-100 and nab-paclitaxel was not beneficial for the treatment of pancreatic cancer.

Yet another strategy to overcome anti-LMB-100 antibody formation in patients has been the use of tofacitinib (a JAK inhibitor), which was previously used in an SS1P study and significantly lowered the production of anti-SS1P antibodies (140). Concurrent administration of LMB-100 and tofacitinib in patients with mesothelin-positive solid tumors has started recently (CTI: NCT04034238). However, according to a recent brief report on the study presented at the annual meeting of the American Society of Clinical Oncology (2021), the combination has yielded no objective responses and even raised certain safety concerns (141). Another strategy has been to combine LMB-100 with monoclonal antibodies against immune checkpoints, CTLA-4 and PD-1. *In vivo* and clinical observations have shown that administration of anti-CTLA-4 as well as anti-PD-1 after treatment with either SS1P or LMB-100 resulted in significant antitumor activity and tumor mass eradication (142–144). In this regard, three clinical trials are ongoing including administration of LMB-100 in combination with ipilimumab (anti-CTLA-4, CTI: NCT04840615) or pembrolizumab (anti-PD-1, CTI: NCT03644550) in malignant mesothelioma, as well as pembrolizumab in NSCLC (CTI: NCT04027946). There are no reports from these studies as of the writing of this review.

### Targeting Epidermal Growth Factor Receptor

EGFRvIII is a variant of epidermal growth factor receptor (EGFR) missing 267 amino acids of the extracellular domain because of an in-frame deletion of exons 2–7 (145, 146). EGFRvIII is not capable of binding to EGF; however, due to its constitutive kinase activity, EGFRvIII-bearing cells grow rapidly and are invasively metastatic (147). Both wild-type EGFR (wtEGFR) and the EGFRvIII are highly expressed on the surface of GBM cells, while normal brain cells express none of these receptors (148). D2C7 is a monoclonal antibody that binds to both wtEGFR and EGFRvIII on the surface of GBM cells and is subsequently internalized. The antibody localizes to tumors expressing the receptors and has thus been investigated as part of a PE-derived IT (149). The targeting moiety was created by fusing the VH and VL domains of D2C7 *via* a (Gly4Ser)3 linker and further stabilized by the introduction of Cys mutations in both domains to form a disulfide bridge (D2C7-scdsFv). To create the IT, D2C7-scdsFv was fused to the PE38KDEL (D2C7-(scdsFv)-PE38KDEL). Cytotoxicity evaluation of the IT showed IC_50_ values ranging from 0.18 to 2.5 ng/ml when assessed on various human epidermoid carcinoma or glioblastoma cell lines. Moreover, the application of the IT in an intracranial tumor murine xenograft overexpressing human wtEGFR increased the survival of the animals by up to 310%. The survival rate was approximately 160% when the IT was administered *via* CED to intracranial tumor xenografts expressing both receptors. CED was also shown to be a reasonable route to provide sufficient concentrations of the IT in tumors (150, 151). Next, the D2C-(scdsFv)-PE38KDEL IT, or D2C7-IT in brief, was subjected to a toxicity study in rats to determine the MTD and the dose level in which no adverse effect (NOAEL) was observed. The rats received different concentrations of the IT up to 0.4 µg/rat *via* CED infusion over 72 h. The MTD was between 0.1 and 0.35 µg/rat, and the NOAEL was 0.05 µg (152, 153). D2C7-IT production was scaled (154) for phase I/II clinical trials involving patients with recurrent GB (CTI: NCT02303678). Phase II is still ongoing, but a brief report at the annual meeting of the American Society of Clinical Oncology (2020) presented preliminary data of the dose-escalation phase I, which involved doses ranging from 440 to 23,354 ng/ml. D7C2-IT was administered in patients (two patients in each dose level) *via* CED, and the patients were examined for DLTs. A dose of 6,920 ng/ml was deemed the most suitable therapeutic dose. Phase II was justified because three patients remained in PR for 28–54 months (56). Another clinical trial combining D7C2-IT with atezolizumab, an anti-PD-1 antibody, has been recently initiated (CTI: NCT04160494) (155) This trial was based on a previous animal study of mice xenografts that received D7C2-IT and either anti-CTLA-4 or anti-PD-1 antibodies. In the animal study, the combination with either antibody resulted in prolonged CRs. Moreover, the surviving animals were resistant to tumor development upon subcutaneous re-challenge, indicating a protective antitumor immunity (156). As of the date of this review, there has been no report on the results of this latest clinical trial.

### Targeting Epithelial Cell Adhesion Molecule

Epithelial cell adhesion molecule (EpCAM, aka CD326) is a transmembrane glycoprotein mediating epithelial-specific intracellular cell adhesion (157). The glycoprotein has been shown to be stably overexpressed on the surface of some malignancies including bladder (158), prostate (159), ovary (160), colorectal, breast, and lung cancers (161–163), while its expression is limited on normal tissues corresponding to the malignancies (162). EpCAM is involved in cell migration, proliferation, and differentiation, as well as cell adhesion. The contribution of EpCAM to proliferation and promotion of tumor growth, and metastasis as well, has made it an attractive antigen for cancer cell-targeted antibodies and ITs (164–166).

Primary attempts to produce an IT targeting EpCAM-positive malignancies were made by chemical conjugation of a full anti-EpCAM antibody, MOC-31, to PE *via* chemical conjugation (167). The IT was effective on human small cell lung cancer (SCLC) *in vitro* and *in vivo* in rat xenograft models (168). A subsequent version of the IT was made by chemical conjugation of the MOC-31 antibody to PE40 (PE_252-613_) and termed MOC31-ETA_252–613_. The IT was cytotoxic to an array of lung cancer cell lines with IC_50_ values ranging from 10 to 3,500 pM. Furthermore, i.p. administration of four doses of the IT measuring 10 or 20 µg every other day in SCLC xenografts showed promising antitumor effects as well as an LD_50_ of 210 µg (169).

To enhance tumor penetration of the IT, a MOC-31 antibody-derived scFv (termed 4D5MOCB) was developed (170) and recombinantly fused to PE_252-608_KDEL, yielding 4D5MOCB-PE_252-608_KDEL (also referred to as 4D5MOCB-PE40KDEL). *In vitro* assessment of 4D5MOCB-PE40KDEL revealed specific cytotoxicity on EpCAM-positive cell lines with IC_50_ values of 0.005–0.2 pM. *In vivo* safety and toxicity evaluation of the IT in mice revealed no organ toxicity. Antitumor activity of the 4D5MOCB-PE40KDEL was verified in nude mice xenografted with SW2 (lung), HT29 (colon), or CAL27 (squamous) carcinoma cell lines. The mice received a total amount of either 45 or 30 µg of 4D5MOCB-PE40KDEL over 3 or 1 weeks, respectively. All animals tolerated the administered doses. Significant tumor regression was observed in all animals that received 45 µg of the IT, with five cases of durable CR (171). Toxicokinetics of the 4D5MOCB-PE40KDEL (now termed VB4-845) was assessed following intratumoral and i.v. administration in rats and cynomolgus monkeys. The MTD was determined to be 1 mg/kg in cynomolgus monkey, and local administration of the IT was safe even after repeated injections. However, in rats administered systemic doses, significant toxicities including vascular leak syndrome (VLS) and multiple organ ischemic necrosis resulting in animal death occurred (50). Despite the effect in rats, and considering the promising antitumor effects of VB4-845, as well as its safety *via* locoregional administration, it was concluded that the IT might be suitable for the treatment of locally accessible malignancies.

A dose-escalation phase I clinical trial involving 20 patients with squamous cell carcinoma of the head and neck (SCCHN) has been conducted. Patients received IT doses ranging at 100–930 μg once weekly for four consecutive weeks. The DLT was grade 3 elevated liver enzyme, and the MTD was determined to be 930 μg/dose. Moreover, a notable reduction in injected tumors size was observed in 10 patients, and four patients showed complete clinical resolution of their tumors (172).

Concurrently, another phase I clinical trial was also performed in patients with EpCAM-positive non-muscle-invasive bladder cancer either refractory to or intolerant of Bacillus Calmette–Guérin (BCG) therapy. The patients received intravesical injections of 0.1–30.16 mg/dose of VB4-845 once weekly for six consecutive doses. Only 20 patients experienced mild VB4-845 administration-related adverse effects; hence, no DLT and, consequently, no MTD was determined. In addition, an overall CR of 39% was observed, most of which occurred at doses higher than 1 mg (173). Thus, since the modality was found to be safe and efficient for the disease, it proceeded to a phase II clinical trial (CTI: NCT00462488) under the name oportuzumab monatox or Vicinium. In that study, 46 patients with non-invasive urothelial carcinoma *in situ* were split into two cohorts (cohorts 1 and 2) and received 30 mg/dose of intravesical Vicinium according to treatment schedules specific for each cohort for up to 1 year. Forty-two patients completed the treatment regimens. All patients tolerated the IT well. No one reported serious adverse effects related to the medication, and no one died during the study. Of the evaluable patients, 44% achieved a CR that was reduced to 40%, 27%, 18%, and 16% when evaluated at 3, 6, 9, and 12 months, respectively. However, seven patients of both cohorts remained with CR for 18–25 months. This study confirmed the safety and efficacy of Vicinium (60). Vicinium is being evaluated in a phase III clinical trial (CTI: NCT04859751) that started on March 23, 2021, and is expected to be completed around December 2023.

Another phase I clinical trial combining Vicinium with durvalumab [a monoclonal anti-PD-L1 antibody (174) to treat patients with high-grade non-muscle-invasive bladder cancer previously treated with BCG (CTI: NCT03258593)] is ongoing. Durvalumab is expected to potentiate Vicinium through an enhancement of immune responses resulting from a PD1–PD-L1 interaction blockade. Yet there is no report on the clinical effects and outcomes of the combination.

## Conclusion

There have been 12 PE–ITs (**Table 3**) with promising effects *in vitro* and in animal studies; however, most could not be translated to the clinic. The inability to transfer the results from the laboratory to the clinic was based on safety or efficacy issues, or both. Safety concerns mostly arise due to non-specific uptake of an IT by non-target cells, which is partly due to the physiologic expression of targeted antigen/receptor by normal cells, though at low levels. Furthermore, immune responses against ITs, which are considered foreign proteins by the immune system, could result in both safety and efficacy issues. Recent publications fully review why the immunogenicity of PE-derived ITs and disease resistance renders this treatment inapplicable in a clinical setting (176, 177); however, given the approval of Moxe by the FDA, and promising clinical effects observed following combination therapies, especially with immune checkpoint inhibitors (178) as well as efforts that are underway to address the safety and efficacy issues (179), PE–ITs remain a promising research area for cancer-specific targeted therapeutic modalities.

**Table 3:** Clinical characteristics of PE–ITs advanced to clinical trials, alone or in combination with other therapeutic agents.

Immunotoxin	MTD	DLT	Dosing	Response	Reference
Moxe (HA22)	50 µg/kg × 6	CLS, refractory hypercalcemia in the setting of active leukemia, hepatobiliary disorder, HUS	A dose of 40 μg/kg by intravenous (i.v.) infusion over 30 min on days 1, 3, and 5 of 28-day cycles for a maximum of six cycles	Significant clinical response in hairy cell leukemia	(79)
IL-13-pe38qqr	0.5 mg/ml in a volume of 72 ml administered by CED for 96 h at a rate of 0.750 ml/h	Symptomatic imaging changes consistent radiographically and histopathologically with a necrotic and inflammatory process	(0.5 mg/ml; total flow rate 0.75 ml/h) was administered over 96 h *via* 2–4 intraparenchymal catheters placed	No significant clinical response	(52)
SS1P (SS1(dsFv)-PE38)	45 µg/kg	Allergic reactions, VLS, pleuritic	25 to 55 µg/kg was administered intravenously over 30 min	A few cases of minor clinical response, but not CR	(116)
LMB-100	140 µg/kg	CLS	40–250 µg/kg of LMB-100 *via* 30-min intravenous infusion on days 1, 3, and 5 of a 21-day cycle	No significant clinical response	(138)
LMB-100+Nab-paclitaxel	65 mg/kg	CLS (edema, urine output decrease)	Received fixed-dose nab-paclitaxel (125 mg/m^2^ on days 1 and 8) with LMB-100 (65 or 100 mg/kg on days 1, 3, and 5) in 21-day cycles for 1–3 cycles	No significant clinical response	(51)
LMB-100+tofacitinib	ND	CLS-related cardiac toxicity and hyponatremia	Tofacitinib 10 mg twice daily on days 1–10 and LMB-100 at 65, 100, or 140 μg/kg on days 4, 6, and 8 of a 21-day cycle	No significant clinical response, inadequate safety	(141)
LMB-100+ ipilimumab	Ongoing	Ongoing	Ongoing	Ongoing	(175)
LMB-100+pembrolizumab	Ongoing	Ongoing	Ongoing	Ongoing	(138)
D2c7-pe38kdel	6,920 ng/ml	Seizure, confusion, pyramidal tract syndrome, cerebral edema, dysphasia	440–23,354 ng/ml	Ongoing	(56)
D2c7-pe38kdel+ atezolizumab	Ongoing	Third-grade ALT elevation, no final report yet	Ongoing	Ongoing	(155)
Oportuzumab monatox or Vicinium	930 μg/dose	Elevated liver enzyme	30 mg/dose once per week for 6 weeks (cohort 1) 30 mg/dose once per week for 12 consecutive weeks (cohort 2)	Significant clinical response, proceeded to phase III (CTI: NCT04859751)	(60)
Cd4-pe40	80 µg/m^2^	Hepatocellular injuries	40, 80, or 160 µg/m^2^ CD4-PE by infusion three to seven times over 10 days	No clinical response (viral infection restarts after treatment stops)	(53)
LMB-2	40 µg/kg every other day for 3 doses	Transaminase elevation, diarrhea, and cardiomyopathy	2 to 63 µg/kg administered intravenously over 30 min on alternate days for three doses (qod × 3)	No significant clinical response as well as progressive disease	(54)
Erb-38	ND	ND	Three doses of erb-38 at 1.0 and 2.0 μg/kg	No clinical response	(55)
LMB-1	75 µg/kg given i.v. three times every other day	Transient postural hypotension and scanty urination	10, 15, 20, 25, 30, 45, 60, 75, 90, and 100 µg/kg	Inadequate safety	(57)
Nbi-3001	6 µg/ml × 40 ml	Transaminase elevation	6 µg/ml × 40 ml, 9 µg/ml × 40 ml, 15 µg/ml × 40 ml, or 9 µg/ml × 100 ml	No significant clinical responses, inadequate safety	(58)
Sgn-10	0.641 mg/m^2^	Gastrointestinal side effects	0.024 to 0.962 mg/m^2^	No clinical responses	(59)
Ovb3-PE	0.5 µg when administered i.p. every other day for a total of 3 or 6 treatments	CNS toxicities	1 to 10 µg/kg	No clinical responses	(61)

CLS, capillary leak syndrome; CR, complete remission; CTI, Clinicaltrials.gov Identifier; DLT, dose-limiting toxicity; HUS, hemolytic uremic syndrome; MTD, maximum tolerated dose; VLS, vascular leak syndrome; PE, Pseudomonas exotoxin A; IT, immunotoxin; CED, convection-enhanced delivery; ALT, alanine aminotransferase; CNS, central nervous system. ND, not defined.

## Author Contributions

SH, MA, and AJ took part in drafting, revising, and preparing the manuscript. The manuscript was finalized and prepared for submission by AJ. All authors contributed to the article and approved the submitted version.

## Conflict of Interest

The authors declare that the research was conducted in the absence of any commercial or financial relationships that could be construed as a potential conflict of interest.

## Publisher’s Note

All claims expressed in this article are solely those of the authors and do not necessarily represent those of their affiliated organizations, or those of the publisher, the editors and the reviewers. Any product that may be evaluated in this article, or claim that may be made by its manufacturer, is not guaranteed or endorsed by the publisher.

## Abbreviations

BBB, blood–brain barrier; BCG, Bacillus Calmette–Guérin; CED, convection-enhanced delivery; CLL, chronic lymphocytic leukemia; CR, complete remission; DIPG, diffuse intrinsic pontine glioma; DLT, dose-limiting toxicity; FDA, United States Food and Drug Administration; GB, glioblastoma; GBM, glioblastoma multiforme; HCL, hairy cell leukemia; HUS, hemolytic uremic syndrome; IT, immunotoxin; i.p., intraperitoneal; i.v., intravenous; MRD, minimal residual disease; MTD, maximum tolerated dose; NHL, non-Hodgkin’s lymphoma; SCCHN, squamous cell carcinoma of the head and neck; SCLC, small cell lung cancer; PE, *Pseudomonas* exotoxin A; PR, partial remission.
