# Supplementary material for: Pseudomonas Exotoxin-Based Immunotoxins: Over Three Decades of Efforts on Targeting Cancer Cells With the Toxin
Source: Front Oncol. 2021 Dec 16;11:781800. doi: 10.3389/fonc.2021.781800 (PMC8716853; doi:10.3389/fonc.2021.781800)
Supplement: Supplementary file 1 [file Table_1.doc]

Supplementary Table 1. A summary of all studies regarding production and evaluation of PE-derived immunotoxins which retrieved from scientific databases. The studies have been sorted according to the “extent of the studies”.

| **Immunotoxin** | **Toxin fragment** | **Targeting moiety** | **Target disease** | **Extent of study** | **Reference** |
| --- | --- | --- | --- | --- | --- |
| Moxetumomab | PE38 | Anti-CD22 | Hairy cell leukaemia | FDA-approved | (Dhillon, 2018) |
| IL-13-PE38QQR | PE38QQr | IL-13 | Glioblastoma multiforme | Clinical trial phase 3 | (Kunwar et al., 2010) |
| CD4-PE40 | PE40 | CD4 | HIV | Clinical trial phase 3 | (Ramachandran et al., 1994) |
| VB4-845  (Oportuzumab monatox or Vicinium) | PE | anti-EpCAM | Squamous cell carcinoma of the head and neck | Clinical trial  Phase 3 | (Kowalski et al., 2012) |
| LMB-100 | PE38 | Anti-Mesothelin | Pancreatic  Adenocarcinoma | Clinical trial phase 1 & 2 | (Alewine et al., 2020) |
| LMB-2 | PE38 | Anti-IL2  (Anti-TAC) | Hematological malignancies | Clinical trial phase 2 | NCT00077922  (Kreitman et al., 2016) |
| NBI-3001 | PE38KDEL | IL4 | Solid tumors | Clinical trial phase 2 | NCT00014677  (Weber et al., 2003) |
| erb-38 | PE38 | Fv portion of MAb e23 | Breast cancer & esophageal cancer | Clinical trial phase 1 | (Pai-Scherf et al., 1999) |
| D2C7-IT | PE38KDEL | EGFR | Glioblastoma | Clinical trial phase 1 | (Desjardins et al., 2020) NCT02303678 |
| LMB-1 | PE38 | B3 | Solid tumors | Clinical trial phase 1 | (Pai et al., 1996) |
| SGN-10 | PE40 | BR96 | Advanced solid tumor | Clinical trial  Phase 1 | (Posey et al., 2002) |
| OVB3- PE | PE | OVB3 | Ovarian cancer | Clinical trial  Phase 1 | (Pai et al., 1991a) |
| IL2-PE40 | PE40 | IL2 | Graft rejection | *in vivo* | (Lorberboum-Galski et al., 1989) |
| TGFc~-PE40 | PE40 | TGF alpha | Various cancers | *in vivo* | (Pai et al., 1991b) |
| TGFα-PE38 | PE38 | TGF alpha | Various cancers | *in vivo* | (Lim et al., 2017) |
| IL6-PE40 and IL6-PE664Glu | PE40 & PE66Glu | IL6 | Hepatocellular carcinoma | *in vivo* | (Siegall et al., 1991) |
| IL6-PE4E-TGF | PE40 | IL6 and TGF | Various cancers | *in vivo* | (Kreitman et al., 1992b) |
| scFv(FRP5)-TGF alphaETA | ETA | TGF alpha  &  ErbB-2 | Various cancers | *in vivo* | (Schmidt and Wels, 1996) |
| anti-TFR-LysPE40 | LysPE40 | anti-TFR | Various cancers | *in vivo* | (Batra et al., 1989) |
| Fab-NlysPE40 | NlysPE40 | anti-TFR (HB21) | Epidermoid and colon carcinoma | *in vivo* | (Debinski and Pastan, 1992) |
| Tfn-PE | PE | Tfn | LOX melanoma | *in vivo* | (Hall et al., 1994) |
| IL6(T23)-PE38KDEL | PE38KDEL | IL6 | Multiple myeloma (MM) | *in vivo* | (Guo et al., 2012) |
| CD25-PE38KDELKQK | PE38KDELKQK | CD25 | Anti-tumor activity | *in vivo* | (Wang et al., 2008) |
| RFT5(SCFV)-ETA' | ETA’ | anti-CD25 | Hodgkin lymphoma | *in vivo* | (Barth et al., 2000a) |
| HRG-PE38KDEL | PE38KDEL | EGFR3 and ErbB2/neu | Various cancers | *in vivo* | (Yang et al., 1998) |
| 425(scFv)-ETA' | ETA | antiEGFR | Pancreatic cancer | *in vivo* | (Bruell et al., 2005) |
| MR1scFvPE38KDEL (MR-1) | PE38KDEL | anti-EGFRvIII (MR1) | Neoplastic meningitis | *in vivo* | (Archer et al., 1999) |
| MOC31-PE, BM7-PE & anti-EGFr-PE | PE | MOC31, BM7 and anti-EGFr | Breast cancer | *in vivo* | (Engebraaten et al., 2000) |
| dEGF4KDEL | PE38KDEL | EGFR and IL-4R | Pancreatic cancer | *in vivo* | (Oh et al., 2012) |
| 806-PE38 | PE38 | anti-EGFR | Breast cancer | *in vivo* | (Simon et al., 2016) |
| VEGF165-PE38 | PE38 | VEGF | Malignant glioma model | *in vivo* | (Hu et al., 2010) |
| C242-PE | PE | C242 | Colon cancer | *in vivo* | (Debinski et al., 1992) |
| C242(Fv)PE38KDEL & C242(Fv)PE38QQR & C242K-PE38QQR & C242rF(ab’)-PE38QQR & C242rF(ab’)-PE38QQRD |  | C242 | Colorectal | *in vivo* | (Debinski and Pastan, 1995) |
| B1(dsFv)-PE33 | PE33 | B1(dsFv) | Various cancers | *in vivo* | (Kuan and Pastan, 1996) |
| Ki-4(scFv)-ETA | ETA | anti-CD30 | Hodgkin tumors | *in vivo* | (Barth et al., 2000b) |
| Anti-CD30-CL1(Fv)-PE38  Anti-CD30-CL2(Fv)-PE38  Anti-CD30-CL2(Fv)PE38KDEL  Anti-CD30-CL4(Fv)-PE38 |  | anti-CD30 | Hodgkin’s disease and anaplastic large cell lymphoma | *in vivo* | (Rozemuller et al., 2001) |
| H22(scFv)-ETA | ETA | anti-CD64 | Chronic cutaneous inflammation | *in vivo* | (Ribbert et al., 2010) |
| TARC-PE38, MC148-PE38, CTACK-PE38 | PE38 | anti-CCR4 | Cutaneous T cell lymphoma/leukemia | *in vivo* | (Baatar et al., 2007) |
| RANTES-PE38 | PE38 | anti-CCR5 | HIV | *in vivo* | (Merriam et al., 2017) |
| IL15-PE293 and IL15MPE293 | PE293 | IL15 | Arthritis | *in vivo* | (Wang et al., 2010) |
| PG001 and PG002 ( VHH6- PE38 and dVHH6PE38) | PE38 | anti-CD7 | Acute lymphoblastic leukemia (T-ALL) and acute myeloid leukemia (AML) | *in vivo* | (Tang et al., 2016) |
| dhuVHH6-PE38, dVHH6PE-LR, and dhuVHH6-PEL | PE | anti-CD7 | Malignant tumors, especially T-cell acute lymphoblastic leukemia | *in vivo* | (Yu et al., 2017) |
| PE-NPs-HER | PE38KDEL | anti-(rhuMAbHER2) | Breast cancer | *in vivo* | (Chen et al., 2008) |
| HER2-affitoxin H | PE38KDEL | anti-HER2 | Various cancers | *in vivo* | (Zielinski et al., 2009) |
| ABD-ZHER2-PE38 | PE38 | HER2 | Her2 positive cancers | *in vivo* | (Guo et al., 2016b) |
| HER2-PE25-X7 | Mutated  PE38 | HER2 | Her2 positive cancers | *in vitro* | (Guo et al., 2016a) |
| 4D5scFv-PE40 | PE40 | Anti HER2 | Her2 positive cancers | *in vivo* | (Sokolova et al., 2015) |
| 4D5scFv-ETA | ETA | anti-HER2 | Ovarian carcinoma | *in vivo* | (Zdobnova et al., 2015) |
| DARPin-PE40 | PE40 | HER2-specific DARPin | Breast cancer | *in vivo* | (Sokolova et al., 2016) |
| DARPin-LoPE | LoPE | HER2-specific DARPin | Human ovarian carcinoma | *in vivo* | (Sokolova et al., 2019) |
| dsFv anti-FRbeta-PE38 | PE38 | anti-FRbeta | Arthritis | *in vivo* | (Nagai et al., 2012) |
| A5-PE40 | PE40 | anti-PSMA | Prostate cancer | *in vivo* | (Wolf et al., 2008) |
| D7-PE40 | PE40 | dsFV anti-PSMA | Prostate cancer | *in vivo* | (Wolf et al., 2010) |
| MRK16-PE | PE | human-specific anti-P-glycoprotein monoclonal antibody | Various cancers | *in vivo* | (Mickisch et al., 1992) |
| LL2-PE38KDEL  LL2-Fab'-PE38KDEL | PE38KDEL | LL2  LL2-Fab' | Burkitt's lymphoma | *in vivo* | (Kreitman et al., 1993) |
| ME20-LysPE40 | PE40 | ME20 | Melanoma | *in vivo* | (Wolff et al., 1995) |
| GnRH-PE66 | PE66 | GnRH | Cancer | *in vivo* | (Nechushtan et al., 1997) |
| Fc(2'-3)-PE40 | PE40 | Fc fragment of mouse IgE | Allergic disorders | *in vivo* | (Fishman and Lorberboum-Galski, 1997) |
| TP-3(scFv)-PE38 & TP-3(dsFv)-PE38 | PE38 | TP-1 (immunoglobulin 2a) and TP-3 (immunoglobulin 2b) | Osteosarcoma | *in vivo* | (Onda et al., 2001) |
| IL-3PE | PE | (IL)-3 | Leukemia | *in vivo* | (Vallera et al., 2003) |
| 8H9(scFv)-PE38  & 8H9(dcFv)-PE38 | PE38 | 8H9 | Breast cancer, osteosarcoma, and neuroblastoma | *in vivo* | (Onda et al., 2004) |
| 9.2.27-PE | PE | Targets highmolecular-weight melanoma-associated antigen (HMW-MAA) | Glioblastoma | *in vivo* | (Hjortland et al., 2004) |
| CD19-ETA | ETA | CD19 | B-lineage leukemia | *in vivo* | (Schwemmlein et al., 2007) |
| Fab2F1PE38KDEL  CAG10PE38KDEL CLA12-PE38KDEL | PE38KDEL | T-Cell Receptor–like Specificity | Melanoma | *in vivo* | (Klechevsky et al., 2008) |
| SMFv-PE38KDEL | PE38KDEL | antibody derived from SM5-1 monoclonal antibody | Melanoma, hepatocellular carcinoma and breast cancer | *in vivo* | (Wang et al., 2007) |
| (PE-NP-S) SMFv-PE38KDEL type I mutant (PE38KDEL-I; Mut-I) | PE38KDEL | SM5 | Hepatocellular carcinoma | *in vivo* | (Gao et al., 2008) |
| scFv35-ETA | ETA | anti-fAChR | Rhabdomyosarcoma | *in vivo* | (Gattenlöhner et al., 2010) |
| dCD133KDEL | PE38KDEL | anti-CD133 | Human head and neck cancer | *in vivo* | (Waldron et al., 2011) |
| F6V-PE38 | PE38 | anti-glycoprotein NMB | Gliomas and melanomas | *in vivo* | (Kuan et al., 2011) |
| SWA11-ZZ-PE38  &  IgG-ZZ-PE38 | PE38 | anti-CD24 | Human colorectal tumors | *in vivo* | (Shapira et al., 2011) |
| EGFATFKDEL 7mut | PE38KDEL | anti-EGF/ ATF | Glioblastoma and its neovasculature in the brain | *in vivo* | (Oh et al., 2011) |
| anti-c-Met/PE38KDEL | PE38KDEL | anti-c-Met | Cancer  (hepatoma) | *in vivo* | (Liu et al., 2013) |
| NZ-1-PE38KDEL  NZ-1-(scdsFv)-PE38KDEL | PE38KDEL | anti-podoplanin (NZ-1) | Brain tumors | *in vivo* | (Chandramohan et al., 2013) |
| rG17PE38 | PE38 | Anti-gastrin/cholecystokinin subtype 2 receptor (CCK2R) | Gastric cancer | *in vivo* | (Song et al., 2013) |
| MSH-PE38KDEL | PE38KDEL | MSH | Melanoma | *in vivo* | (Hui et al., 2014) |
| HM1.24-ETA' | ETA | CD317 | Myeloma and leukemia | *in vivo* | (Staudinger et al., 2014) |
| rCCK8PE38 | PE38 | anti-CCK-R | Colon cancer | *in vivo* | (Gao et al., 2015a) |
| YP7-PE38 | PE38 | anti-glypican-3, HN3 and YP7 | Liver tumour | *in vivo* | (Gao et al., 2015b) |
| Fcε-PE40 | PE40 | Anti-mast cell | Colon cancer | *in vivo* | (Wang et al., 2016) |
| HN3-T20  HN3-PE38  HN3-ABD-T20  HN3-T19  HN3-M11  HN3-mPE24 | PE38 | anti-GPC3 | Liver cancer | *in vivo* | (Fleming et al., 2020) |
| chDMB5F3:ZZ-PE38 | ZZ-PE38 | anti-MUC1 SEA | Various cancers | *in vivo* | (Pichinuk et al., 2020) |
| Z142X | PE38KDEL | ZEBV LMP-2 | Nasopharyngeal carcinoma | *in vivo* | (Zhu et al., 2020) |
| T22-PE24-H6 | PE24 | T22 | Large b-cell lymphoma | *in vivo* | (Falgàs et al., 2020) |
| 2E4- PE38 | PE38 | 2E4 | Various cancers | *in vivo* | (Onda et al., 2019) |
| LMB70  LMB75 | PE24 | anti-BCMA-fab  anti-BCMA-disulfidestabilized (ds)-Fv | Myeloma | *in vivo* | (Shancer et al., 2019) |
| αPD-1–ABD–PE | PE | single-chain variable fragment (scFv) of the PD-1 antibody (αPD-1) | Autoimmune diseases | *in vivo* | (Zhao et al., 2019) |
| HN3-mPE24 | mPE24 | Bivalent NH3 | Liver cancer | *in vivo* | (Wang et al., 2017) |
| B1(Fv)PE38 | PE38 | B1(Fv) | Various cancers | *in vivo* | (Benhar and Pastan, 1995) |
| B1(dsFv)PE38 | PE38 | B1(dsFv) | Various cancers | *in vivo* | (Benhar and Pastan, 1995) |
| 1H10-PE | PE | 1H10 | Cervical carcinoma | *in vivo* | (Roffler et al., 1991) |
| PE37/ TGF-a | PE37 | TGF alpha | Various cancers | *in vitro* | (Theuer et al., 1992) |
| cysPE35/TGF-alpha | cysPE35 | TGF alpha | Cells with the human transferrin receptor | *in vitro* | (Theuer et al., 1993b) |
| PE35/TGFα-KDEL | PE35 | TGF alpha KDEL | Bladder cancer | *in vitro* | (Theuer et al., 1993a) |
| Anti-TFR-PE | PE | anti-TFR (HB21) | Ovarian carcinoma | *in vitro* | (Pirker et al., 1985) |
| DTM1-E6 sFv-PE40 COOH | PE40 | Anti-TNF | Various cancers | *in vitro* | (Nicholls et al., 1993) |
| G28-5 sFv-PE40 | PE40 | Anti-CD40 | Various cancers | *in vitro* | (Francisco et al., 1996) |
| IL6-PE | PE | IL-6 | Prostate | *in vitro* | (Siegall et al., 1990b) |
| IL-6-PE40 | PE40 | IL-6 | Myeloma | *in vitro* | (Siegall et al., 1990a) |
| IL6-PE66 | PE66 | IL-6 | Human prostate cancer  Hepatocellular carcinoma | *in vitro* | (Siegall et al., 1990b) |
| RFT5(scFv)-ETA' | ETA | anti-CD25 | Hodgkin lymphoma | *in vitro* | (Barth et al., 1998) |
| huscFv-PE25 | PE25KDEL | anti-EGFR huscFv | Various cancers | *in vitro* | (Akbari et al., 2017) |
| VEGF121-PE38 | PE38KDEL | VEGF121 | Solid tumors | *in vitro* | (Langari et al., 2017) |
| VGRNb-PE | PE38 | VEGFR2-specific Nanobody | Transplastomic lettuce (Lactuca sativa L.) | *in vitro* | (Mirzaee et al., 2018) |
| CD30L-ETA' | ETA' | CD30L | Hodgkin's disease | *in vitro* | (Barth et al., 1999) |
| HeFi-I-PE38  T6-PE38  T13-PE38  T14-A-PE38  T14-B-PE38  T24-PE38  T25-PE38 | PE38 | anti-CD30 | Hodgkin’s disease and anaplastic large cell lymphoma | *in vitro* | (Nagata et al., 2002) |
| CD7-ETA & MCSP-ETA | ETA | CD7-specific | Acute leukemia | *in vitro* | (Peipp et al., 2002) |
| Trastuzumab-PE24 | PE24 | (Her2)-targeting IgG, trastuzumab | Breast cancer | *in vitro* | (Lee et al., 2019) |
| CD123-ETA’ and  bsscFv [123 · ds16] | ETA | CD123 | Myeloid leukaemia cells | *in vitro* | (Stein et al., 2010) |
| 26292(Fv)-PE38 & 32716(Fv)-PE38 & 32701(Fv)-PE38 | PE38 | anti-CD123 | Acute myeloid leukemia | *in vitro* | (Du et al., 2007) |
| J591scFvPE38QQR ( J591PE) | PE38QQR | anti-PSMA | Prostate cancer | *in vitro* | (Baiz et al., 2013) |
| MAB-PE | PE | Breast tumor selective ab (MAB) | Breast tumor | *in vitro* | (Bjorn et al., 1986) |
| NR-LU-10-PE | PE | pancarcinoma monoclonal antibody (NR-LU-10), | Breast cancer | *in vitro* | (Bjorn et al., 1990) |
| 0.5ß-PE (PE-ß) | PE | monoclonal antibody (0.5ß) against the exterior envelope  protein (gpl20) of  HIV2 | HIV | *in vitro* | (Matsushita et al., 1990) |
| (9.2.27 or NR-ML-05)-PE | PE | 9.2.27 or NR-ML-05 (bind to different epitopes on the melanoma-associated antigen p250) | Melanoma | *in vitro* | (Godal et al., 1992) |
| Mik-beta 1(Fv)-PE40 | PE40 | Mik-beta 1 | Various cancers | *in vitro* | (Kreitman et al., 1992a) |
| K1-PE | PE | K1 is a monoclonal antibody that reacts with a cell surface antigen (CAK1) | Ovarian cancer | *in vitro* | (Chang et al., 1992) |
| PR1(Fv)-PE38KDEL | PE38KDEL | PR1(Fv) | Prostate adenocarcinoma | *in vitro* | (Brinkmann et al., 1993) |
| SEN7-PE | PE | SEN7 | Sclc | *in vitro* | (Zangemeister-Wittke et al., 1994) |
| HAR-TX β2 | PE40 | heregulin-β2 | Carcinoma | *in vitro* | (Siegall et al., 1995) |
| rhIL-9-ETA' | ETA | IL9 | Aml | *in vitro* | (Klimka et al., 1996) |
| GM-CSF-PE40 | PE40 | GM-CSF | Myeloid leukaemias | *in vitro* | (O’Brien et al., 1997) |
| hMN14(Fv)-PE40 | PE40 | (hMN14(Fv)) | Colorectal carcinoma | *in vitro* | (Akamatsu et al., 1998) |
| CEA/PE38/KDEL | PE38KDEL | CEA | Colorectal carcinoma | *in vitro* | (Yang et al., 2004) |
| E4-PE35KDEL  E4(Fv)-PE38KDEL | PE35KDEL | E4 | Various cancers | *in vitro* | (Essand and Pastan, 1998) |
| 3B3(Fv)-PE38 | PE38 | anti-gp120 antibody | HIV | *in vitro* | (Bera et al., 1998) |
| MBP-PE 40 | PE40 | myelin basic protein | Experimental autoimmune encephalomyelitis (MS) | *in vitro* | (Brenner et al., 1999) |
| MR1(Fv)(VHS98P-T99Y)-PE38 & MR1-1(Fv)-PE38 | PE38 | MR1 mutants | Glioblastomas | *in vitro* | (Beers et al., 2000) |
| Dsg3deltaN1-PE40-KDEL & PE3 7-Dsg3deltaN1-KDEL | PE40KDEL | anti-desmoglein 3 (Dsg3) | Pemphigus vulgaris  (PV)  {autoimmune  Blistering disease} | *in vitro* | (Proby et al., 2000) |
| ATF-PE38  & ATF-PE38KDEL | PE38  PE38KDEL | the amino-terminal fragment (ATF) of human urokinase which binds to (2)macroglobulin receptor ((2)MR) | Various cancers | *in vitro* | (Rajagopal and Kreitman, 2000) |
| 14.18(scFv)-ETA' | ETA | anti-GD2 | Neuroblastoma | *in vitro* | (Tur et al., 2001) |
| scFv(UCHT1)-PE38 scFv(BC3)-PE38  scFv(SP34)-PE38  scFv-(145-2C11)-PE38 | PE38 | anti-CD3 | Various cancers | *in vitro* | (Hexham et al., 2001) |
| 9F11(Fv)-PE38 | PE38 | 9F11 monoclonal antibody (IgG1; k light chain)  {Pgp-expressing MDR carcinoma cells lines} | Various cancers | *in vitro* | (Niv et al., 2001) |
| C7(scFv)-PE | PE | anti-Carcinoembryonic antigen (CEA) | Various cancers | *in vitro* | (Kim, 2003) |
| A5scFv-PE38 | PE38 | A5 (targets the extracellular N-terminus of the human MRP1 expressed on the surface of live tumor cells) | Various cancers | *in vitro* | (Binyamin et al., 2005) |
| CD33-ETA | ETA | anti-CD33 | Myeloid leukaemia | *in vitro* | (Schwemmlein et al., 2006) |
| B7-2-L-PE40KDEL | PE40KDEL | Anti- B7:CD28 | Graft-versus-host or host-versus-graft diseases | *in vitro* | (Xi et al., 2006) |
| scFv(MUC1)-ETA | ETA | anti-MUC1 | Breast cancers | *in vitro* | (Singh et al., 2007)v |
| ZZ-PE38 | PE38 | Anti IgG  Fc-binding ZZ domain, derived from Streptococcal protein A | Various cancers | *in vitro* | (Mazor et al., 2007) |
| MCSP-ETA' | ETA | anti-melanoma associated chondroitin sulfate proteoglycan (MCSP) | Melanoma | *in vitro* | (Schwenkert et al., 2008) |
| HMGN2-PE38KDEL | PE38KDEL | HMGN2 | Various cancers | *in vitro* | (Xiong et al., 2008) |
| MOG-ETA′ | ETA | myelin oligodendrocyte glycoprotein (MOG) | Autoimmune disorders | *in vitro* | (Nachreiner et al., 2008) |
| EMI405(scFv)-ETA’, and 425(scFv)-ETA’ | ETA | anti-AML blast cells | Acute myeloid leukemia | *in vitro* | (Fitting et al., 2015) |
| 25-D1.16 (Fab)-PE38 | PE38 | anti-SIINFEKL | Rabies virus | *in vitro* | (Mareeva et al., 2010) |
| alpha-kappa-ETA' | ETA | anti-kappa | Various cancers | *in vitro* | (Kellner et al., 2011) |
| YC15-PE38 | PE38 | anti-KSHV glycoprotein H (gH) | Kaposi's sarcoma | *in vitro* | (Cai and Berger, 2011) |
| 2014-PE38 | PE38 | anti- the viral gpK8.1A envelope glycoprotein | Kaposi's sarcomaassociated herpesvirus lytically infection | *in vitro* | (Chatterjee et al., 2012) |
| BT-1 | PE38 | anti-ROR1 | B cell malignancies and other cancers | *in vitro* | (Baskar et al., 2012) |
| CPEETA | Clostridium perfringens enterotoxin (CPE)  + ETA | anti-claudin4 | Head and neck and breast cancers | *in vitro* | (Hashimi et al., 2013) |
| hscFv-ETA’ | ETA | humanized single chain variable fragment (hscFv) | Chronic myeloid lekemia | *in vitro* | (Zhu et al., 2013) |
| A300E–ETA | alpha  FcETA | anti-ADAM17 | Breast cancer | *in vitro* | (Trad et al., 2013) |
| alphaMCSP-ETA' | ETA | anti-CSPG4 | Rhabdomyosarcoma | *in vitro* | (Brehm et al., 2014) |
| anti–FCRL-5 immunotoxin (F56-IT and F25-IT) | PE38 | anti-FCRL-5 | Hepatitis c virus– associated  Mixed  Cryoglobulinemia  Vasculitis | *in vitro* | (Terrier et al., 2014) |
| PiPP-PE38 | PE38 | scFv(PiPP) anti-human chorionic gonadotropin (hCG/hCGbeta) | Cancers | *in vitro* | (Nand et al., 2015) |
| CD89-ETA' | ETA | Fc-alphaRI (CD89) | Myeloid leukemia | *in vitro* | (Mladenov et al., 2015) |
| scFv13-ETA' | ETA | anti-CD13 | Various cancers | *in vitro* | (Grieger et al., 2017) |
| PSCA(scFv)-ETA' | ETA | anti-PSCA | Prostate cancer | *in vitro* | (Kessler et al., 2017) |
| BoScFv-PE38 | PE38 | anti-BoHV-1 | Bohv-1 infection | *in vitro* | (Xu et al., 2018) |
| PE38KDEL-1F12 | PE38KDEL | anti-EPHA2 | Breast cancer | *in vitro* | (Rezaie et al., 2020) |
| C-CPE-ETA' | ETA | anti-CLDN-3, 4 | Ovarian cancer | *in vitro* | (Yao et al., 2010) |
| IL-10(18-57)-PE40 | PE40 | IL10 | Cancers | *in vitro* | (Peng et al., 2006) |

References:

Akamatsu, Y., Murphy, J. C., Nolan, K. F., Thomas, P., Kreitman, R. J., Leung, S. O., et al. (1998). A single-chain immunotoxin against carcinoembryonic antigen that suppresses growth of colorectal carcinoma cells. *Clin. Cancer Res.* 4, 2825–2832.

Akbari, B., Farajnia, S., Zarghami, N., Mahdieh, N., Rahmati, M., Khosroshahi, S. A., et al. (2017). Construction, expression, and activity of a novel immunotoxin comprising a humanized antiepidermal growth factor receptor scFv and modified Pseudomonas aeruginosa exotoxin A. *Anticancer. Drugs* 28, 263–270. doi:10.1097/CAD.0000000000000452.

Alewine, C., Ahmad, M., Peer, C. J., Hu, Z. I., Lee, M.-J., Yuno, A., et al. (2020). Phase I/II Study of the Mesothelin-targeted Immunotoxin LMB-100 with Nab-Paclitaxel for Patients with Advanced Pancreatic Adenocarcinoma. *Clin. Cancer Res.* 26, 828–836. doi:10.1158/1078-0432.CCR-19-2586.

Archer, G. E., Sampson, J. H., Lorimer, I. A., McLendon, R. E., Kuan, C. T., Friedman, A. H., et al. (1999). Regional treatment of epidermal growth factor receptor vIII-expressing neoplastic meningitis with a single-chain immunotoxin, MR-1. *Clin. Cancer Res.* 5, 2646–2652.

Baatar, D., Olkhanud, P., Newton, D., Sumitomo, K., and Biragyn, A. (2007). CCR4-expressing T cell tumors can be specifically controlled via delivery of toxins to chemokine receptors. *J. Immunol.* 179, 1996–2004. doi:10.4049/jimmunol.179.3.1996.

Baiz, D., Hassan, S., Choi, Y. A., Flores, A., Karpova, Y., Yancey, D., et al. (2013). Combination of the PI3K inhibitor ZSTK474 with a PSMA-targeted immunotoxin accelerates apoptosis and regression of prostate cancer. *Neoplasia* 15, 1172–1183. doi:10.1593/neo.13986.

Barth, S., Huhn, M., Matthey, B., Schnell, R., Tawadros, S., Schinkothe, T., et al. (2000a). Recombinant anti-CD25 immunotoxin RFT5(SCFV)-ETA’ demonstrates successful elimination of disseminated human Hodgkin lymphoma in SCID mice. *Int. J. cancer* 86, 718–724. doi:10.1002/(sici)1097-0215(20000601)86:5<718::aid-ijc18>3.0.co;2-n.

Barth, S., Huhn, M., Matthey, B., Tawadros, S., Schnell, R., Schinkothe, T., et al. (2000b). Ki-4(scFv)-ETA’, a new recombinant anti-CD30 immunotoxin with highly specific cytotoxic activity against disseminated Hodgkin tumors in SCID mice. *Blood* 95, 3909–3914.

Barth, S., Huhn, M., Wels, W., Diehl, V., and Engert, A. (1998). Construction and in vitro evaluation of RFT5(scFv)-ETA’, a new recombinant single-chain immunotoxin with specific cytotoxicity toward CD25+ Hodgkin-derived cell lines. *Int. J. Mol. Med.* 1, 249–256. doi:10.3892/ijmm.1.1.249.

Barth, S., Matthey, B., Huhn, M., Diehl, V., and Engert, A. (1999). CD30L-ETA’: a new recombinant immunotoxin based on the CD30 ligand for possible use against human lymphoma. *Cytokines. Cell. Mol. Ther.* 5, 69–78.

Baskar, S., Wiestner, A., Wilson, W. H., Pastan, I., and Rader, C. (2012). Targeting malignant B cells with an immunotoxin against ROR1. *MAbs* 4, 349–361. doi:10.4161/mabs.19870.

Batra, J. K., Jinno, Y., Chaudhary, V. K., Kondo, T., Willingham, M. C., FitzGerald, D. J., et al. (1989). Antitumor activity in mice of an immunotoxin made with anti-transferrin receptor and a recombinant form of Pseudomonas exotoxin. *Proc. Natl. Acad. Sci. U. S. A.* 86, 8545–8549. doi:10.1073/pnas.86.21.8545.

Beers, R., Chowdhury, P., Bigner, D., and Pastan, I. (2000). Immunotoxins with increased activity against epidermal growth factor receptor vIII-expressing cells produced by antibody phage display. *Clin. Cancer Res.* 6, 2835–2843.

Benhar, I., and Pastan, I. (1995). Characterization of B1 ( Fv ) PE38 and B1 ( dsFv ) PE38 : Single-chain and disulfide- stabilized Fv immunotoxins with increased activity that cause complete remissions of established human c ... Characterization and That Cause Complete Xenografts Remissio. 1.

Bera, T. K., Kennedy, P. E., Berger, E. A., Barbas, C. F. 3rd, and Pastan, I. (1998). Specific killing of H-infected lymphocytes by a recombinant immunotoxin directed against the HIV-1 envelope glycoprotein. *Mol. Med.* 4, 384–391.

Binyamin, L., Assaraf, Y. G., and Reiter, Y. (2005). Probing ATP-dependent conformational changes in the multidrug resistance protein 1 (MRP1/ABCC1) in live tumor cells with a novel recombinant single-chain Fv antibody targeted to the extracellular N-terminus. *Int. J. cancer* 116, 703–709. doi:10.1002/ijc.21124.

Bjorn, M. J., Groetsema, G., and Scalapino, L. (1986). Antibody-Pseudomonas exotoxin A conjugates cytotoxic to human breast cancer cells in vitro. *Cancer Res.* 46, 3262–3267.

Bjorn, M. J., Manger, R., Sivam, G., Morgan, A. C. J., and Torok-Storb, B. (1990). Selective elimination of breast cancer cells from human bone marrow using an antibody-Pseudomonas exotoxin A conjugate. *Cancer Res.* 50, 5992–5996.

Brehm, H., Niesen, J., Mladenov, R., Stein, C., Pardo, A., Fey, G., et al. (2014). A CSPG4-specific immunotoxin kills rhabdomyosarcoma cells and binds to primary tumor tissues. *Cancer Lett.* 352, 228–235. doi:10.1016/j.canlet.2014.07.006.

Brenner, T., Steinberger, I., Soffer, D., Beraud, E., Ben-Nun, A., and Lorberboum-Galski, H. (1999). A novel antigen-toxin chimeric protein: myelin basic protein-pseudomonas exotoxin (MBP-PE 40) for treatment of experimental autoimmune encephalomyelitis. *Immunol. Lett.* 68, 403–410. doi:10.1016/s0165-2478(99)00089-9.

Brinkmann, U., Gallo, M., Brinkmann, E., Kunwar, S., and Pastan, I. (1993). A recombinant immunotoxin that is active on prostate cancer cells and that is composed of the Fv region of monoclonal antibody PR1 and a truncated form of Pseudomonas exotoxin. *Proc. Natl. Acad. Sci. U. S. A.* 90, 547–551. doi:10.1073/pnas.90.2.547.

Bruell, D., Bruns, C. J., Yezhelyev, M., Huhn, M., Muller, J., Ischenko, I., et al. (2005). Recombinant anti-EGFR immunotoxin 425(scFv)-ETA’ demonstrates anti-tumor activity against disseminated human pancreatic cancer in nude mice. *Int. J. Mol. Med.* 15, 305–313.

Cai, Y., and Berger, E. A. (2011). An immunotoxin targeting the gH glycoprotein of KSHV for selective killing of cells in the lytic phase of infection. *Antiviral Res.* 90, 143–150. doi:10.1016/j.antiviral.2011.03.175.

Chandramohan, V., Bao, X., Kato Kaneko, M., Kato, Y., Keir, S. T., Szafranski, S. E., et al. (2013). Recombinant anti-podoplanin (NZ-1) immunotoxin for the treatment of malignant brain tumors. *Int. J. cancer* 132, 2339–2348. doi:10.1002/ijc.27919.

Chang, K., Pai, L. H., Batra, J. K., Pastan, I., and Willingham, M. C. (1992). Characterization of the antigen (CAK1) recognized by monoclonal antibody K1 present on ovarian cancers and normal mesothelium. *Cancer Res.* 52, 181–186.

Chatterjee, D., Chandran, B., and Berger, E. A. (2012). Selective killing of Kaposi’s sarcoma-associated herpesvirus lytically infected cells with a recombinant immunotoxin targeting the viral gpK8.1A envelope glycoprotein. *MAbs* 4, 233–242. doi:10.4161/mabs.4.2.19262.

Chen, H., Gao, J., Lu, Y., Kou, G., Zhang, H., Fan, L., et al. (2008). Preparation and characterization of PE38KDEL-loaded anti-HER2 nanoparticles for targeted cancer therapy. *J. Control. Release* 128, 209–216. doi:10.1016/j.jconrel.2008.03.010.

Debinski, W., Karlsson, B., Lindholm, L., Siegall, C. B., Willingham, M. C., FitzGerald, D., et al. (1992). Monoclonal antibody C242-Pseudomonas exotoxin A. A specific and potent immunotoxin with antitumor activity on a human colon cancer xenograft in nude mice. *J. Clin. Invest.* 90, 405–411. doi:10.1172/JCI115875.

Debinski, W., and Pastan, I. (1992). Monovalent immunotoxin containing truncated form of Pseudomonas exotoxin as potent antitumor agent. *Cancer Res.* 52, 5379–5385.

Debinski, W., and Pastan, I. (1995). Recombinant F(ab’) C242-Pseudomonas exotoxin, but not the whole antibody-based immunotoxin, causes regression of a human colorectal tumor xenograft. *Clin. Cancer Res.* 1, 1015–1022.

Desjardins, A., Randazzo, D., Chandramohan, V., Peters, K. B., Johnson, M. O., Threatt, S., et al. (2020). Phase I trial of D2C7 immunotoxin (D2C7-IT) administered intratumorally via convection-enhanced delivery (CED) for recurrent malignant glioma (MG). *J. Clin. Oncol.* 38, 2566. doi:10.1200/JCO.2020.38.15_suppl.2566.

Dhillon, S. (2018). Moxetumomab Pasudotox: First Global Approval. *Drugs* 78, 1763–1767. doi:10.1007/s40265-018-1000-9.

Du, X., Ho, M., and Pastan, I. (2007). New immunotoxins targeting CD123, a stem cell antigen on acute myeloid leukemia cells. *J. Immunother.* 30, 607–613. doi:10.1097/CJI.0b013e318053ed8e.

Engebraaten, O., Sivam, G., Juell, S., and Fodstad, O. (2000). Systemic immunotoxin treatment inhibits formation of human breast cancer metastasis and tumor growth in nude rats. *Int. J. cancer* 88, 970–976. doi:10.1002/1097-0215(20001215)88:6<970::aid-ijc21>3.0.co;2-q.

Essand, M., and Pastan, I. (1998). Anti-prostate immunotoxins: cytotoxicity of E4 antibody-Pseudomonas exotoxin constructs. *Int. J. cancer* 77, 123–127. doi:10.1002/(sici)1097-0215(19980703)77:1<123::aid-ijc19>3.0.co;2-f.

Falgàs, A., Pallarès, V., Serna, N., Sánchez-García, L., Sierra, J., Gallardo, A., et al. (2020). Selective delivery of T22-PE24-H6 to CXCR4(+) diffuse large B-cell lymphoma cells leads to wide therapeutic index in a disseminated mouse model. *Theranostics* 10, 5169–5180. doi:10.7150/thno.43231.

Fishman, A., and Lorberboum-Galski, H. (1997). Targeted elimination of cells expressing the high-affinity receptor for IgE (Fc epsilon RI) by a Pseudomonas exotoxin-based chimeric protein. *Eur. J. Immunol.* 27, 486–494. doi:10.1002/eji.1830270220.

Fitting, J., Blume, T., Ten Haaf, A., Blau, W., Gattenlohner, S., Tur, M. K., et al. (2015). Phage display-based generation of novel internalizing antibody fragments for immunotoxin-based treatment of acute myeloid leukemia. *MAbs* 7, 390–402. doi:10.1080/19420862.2015.1007818.

Fleming, B. D., Urban, D. J., Hall, M. D., Longerich, T., Greten, T. F., Pastan, I., et al. (2020). Engineered Anti-GPC3 Immunotoxin, HN3-ABD-T20, Produces Regression in Mouse Liver Cancer Xenografts Through Prolonged Serum Retention. *Hepatology* 71, 1696–1711. doi:10.1002/hep.30949.

Francisco, J. A., Kiener, P. A., Moran-Davis, P., Ledbetter, J. A., and Siegall, C. B. (1996). Cytokine activation sensitizes human monocytic and endothelial cells to the cytotoxic effects of an anti-CD40 immunotoxin. *J. Immunol.* 157, 1652–1658.

Gao, J., Kou, G., Chen, H., Wang, H., Li, B., Lu, Y., et al. (2008). Treatment of hepatocellular carcinoma in mice with PE38KDEL type I mutant-loaded poly(lactic-co-glycolic acid) nanoparticles conjugated with humanized SM5-1 F(ab’) fragments. *Mol. Cancer Ther.* 7, 3399–3407. doi:10.1158/1535-7163.MCT-08-0514.

Gao, S., Song, J., Chen, F., Wang, Q., Liu, X., Ren, H., et al. (2015a). A novel immunotoxin - rCCK8PE38 targeting of CCK-R overexpressed colon cancers. *J. Drug Target.* 23, 462–468. doi:10.3109/1061186X.2015.1009073.

Gao, W., Tang, Z., Zhang, Y. F., Feng, M., Qian, M., Dimitrov, D. S., et al. (2015b). Immunotoxin targeting glypican-3 regresses liver cancer via dual inhibition of Wnt signalling and protein synthesis. *Nat. Commun.* 6. doi:10.1038/ncomms7536.

Gattenlöhner, S., Jörissen, H., Huhn, M., Vincent, A., Beeson, D., Tzartos, S., et al. (2010). A human recombinant autoantibody-based immunotoxin specific for the fetal acetylcholine receptor inhibits rhabdomyosarcoma growth in vitro and in a murine transplantation model. *J. Biomed. Biotechnol.* 2010, 187621. doi:10.1155/2010/187621.

Godal, A., Kumle, B., Pihl, A., Juell, S., and Fodstad, O. (1992). Immunotoxins directed against the high-molecular-weight melanoma-associated antigen. Identification of potent antibody-toxin combinations. *Int. J. cancer* 52, 631–635. doi:10.1002/ijc.2910520423.

Grieger, E., Gresch, G., Niesen, J., Woitok, M., Barth, S., Fischer, R., et al. (2017). Efficient targeting of CD13 on cancer cells by the immunotoxin scFv13-ETA’ and the bispecific scFv [13xds16]. *J. Cancer Res. Clin. Oncol.* 143, 2159–2170. doi:10.1007/s00432-017-2468-5.

Guo, D.-J., Han, J.-S., Li, Y.-S., Liu, Z.-S., Lu, S.-Y., and Ren, H.-L. (2012). In vitro and in vivo antitumor effects of the recombinant immunotoxin IL6(T23)-PE38KDEL in multiple myeloma. *Oncol. Lett.* 4, 311–318. doi:10.3892/ol.2012.733.

Guo, R., Cao, L., Guo, W., Liu, H., Xu, H., Fang, Q., et al. (2016a). HER2-targeted immunotoxins with low nonspecific toxicity and immunogenicity. *Biochem. Biophys. Res. Commun.* 475, 93–99. doi:10.1016/j.bbrc.2016.05.044.

Guo, R., Guo, W., Cao, L., Liu, H., Liu, J., Xu, H., et al. (2016b). Fusion of an albumin-binding domain extends the half-life of immunotoxins. *Int. J. Pharm.* 511, 538–549. doi:10.1016/j.ijpharm.2016.07.046.

Hall, W. A., Myklebust, A., Godal, A., Nesland, J. M., and Fodstad, O. (1994). In vivo efficacy of intrathecal transferrin-Pseudomonas exotoxin A immunotoxin against LOX melanoma. *Neurosurgery* 34, 646–649. doi:10.1227/00006123-199404000-00012.

Hashimi, S. M., Yu, S., Alqurashi, N., Ipe, D. S., and Wei, M. Q. (2013). Immunotoxin-mediated targeting of claudin-4 inhibits the proliferation of cancer cells. *Int. J. Oncol.* 42, 1911–1918. doi:10.3892/ijo.2013.1881.

Hexham, J. M., Dudas, D., Hugo, R., Thompson, J., King, V., Dowling, C., et al. (2001). Influence of relative binding affinity on efficacy in a panel of anti-CD3 scFv immunotoxins. *Mol. Immunol.* 38, 397–408. doi:10.1016/s0161-5890(01)00070-0.

Hjortland, G. O., Garman-Vik, S. S., Juell, S., Olsen, O. E., Hirschberg, H., Fodstad, O., et al. (2004). Immunotoxin treatment targeted to the high-molecular-weight melanoma-associated antigen prolonging the survival of immunodeficient rats with invasive intracranial human glioblastoma multiforme. *J. Neurosurg.* 100, 320–327. doi:10.3171/jns.2004.100.2.0320.

Hu, C., Ji, H., Chen, S., Zhang, H., Wang, B., Zhou, L., et al. (2010). Investigation of a plasmid containing a novel immunotoxin VEGF165-PE38 gene for antiangiogenic therapy in a malignant glioma model. *Int. J. cancer* 127, 2222–2229. doi:10.1002/ijc.25217.

Hui, Q., Ma, J., Song, J., Liu, Z., Ren, H., Jiang, W., et al. (2014). In vitro and in vivo studies of antitumor effects of the recombinant immunotoxin MSH-PE38KDEL on melanoma. *Neoplasma* 61, 392–400. doi:10.4149/neo_2014_048.

Kellner, C., Bleeker, W. K., Lammerts van Bueren, J. J., Staudinger, M., Klausz, K., Derer, S., et al. (2011). Human kappa light chain targeted Pseudomonas exotoxin A--identifying human antibodies and Fab fragments with favorable characteristics for antibody-drug conjugate development. *J. Immunol. Methods* 371, 122–133. doi:10.1016/j.jim.2011.06.023.

Kessler, C., Pardo, A., Tur, M. K., Gattenlohner, S., Fischer, R., Kolberg, K., et al. (2017). Novel PSCA targeting scFv-fusion proteins for diagnosis and immunotherapy of prostate cancer. *J. Cancer Res. Clin. Oncol.* 143, 2025–2038. doi:10.1007/s00432-017-2472-9.

Kim, S.-H. (2003). Expression and purification of recombinant immunotoxin--a fusion protein stabilizes a single-chain Fv (scFv) in denaturing condition. *Protein Expr. Purif.* 27, 85–89. doi:10.1016/s1046-5928(02)00539-9.

Klechevsky, E., Gallegos, M., Denkberg, G., Palucka, K., Banchereau, J., Cohen, C., et al. (2008). Antitumor activity of immunotoxins with T-cell receptor-like specificity against human melanoma xenografts. *Cancer Res.* 68, 6360–6367. doi:10.1158/0008-5472.CAN-08-0928.

Klimka, A., Barth, S., Drillich, S., Wels, W., van Snick, J., Renauld, J. C., et al. (1996). A deletion mutant of Pseudomonas exotoxin-A fused to recombinant human interleukin-9 (rhIL-9-ETA’) shows specific cytotoxicity against IL-9-receptor-expressing cell lines. *Cytokines Mol. Ther.* 2, 139–146.

Kowalski, M., Guindon, J., Brazas, L., Moore, C., Entwistle, J., Cizeau, J., et al. (2012). A phase II study of oportuzumab monatox: An immunotoxin therapy for patients with noninvasive urothelial carcinoma in situ previously treated with bacillus Calmette-Guérin. *J. Urol.* 188, 1712–1718. doi:10.1016/j.juro.2012.07.020.

Kreitman, R. J., Hansen, H. J., Jones, A. L., FitzGerald, D. J., Goldenberg, D. M., and Pastan, I. (1993). Pseudomonas exotoxin-based immunotoxins containing the antibody LL2 or LL2-Fab’ induce regression of subcutaneous human B-cell lymphoma in mice. *Cancer Res.* 53, 819–825.

Kreitman, R. J., Schneider, W. P., Queen, C., Tsudo, M., Fitzgerald, D. J., Waldmann, T. A., et al. (1992a). Mik-beta 1(Fv)-PE40, a recombinant immunotoxin cytotoxic toward cells bearing the beta-chain of the IL-2 receptor. *J. Immunol.* 149, 2810–2815.

Kreitman, R. J., Siegall, C. B., Chaudhary, V. K., FitzGerald, D. J., and Pastan, I. (1992b). Properties of chimeric toxins with two recognition domains: interleukin 6 and transforming growth factor alpha at different locations in Pseudomonas exotoxin. *Bioconjug. Chem.* 3, 63–68. doi:10.1021/bc00013a010.

Kreitman, R. J., Stetler-Stevenson, M., Jaffe, E. S., Conlon, K. C., Steinberg, S. M., Wilson, W., et al. (2016). Complete Remissions of Adult T-cell Leukemia with Anti-CD25 Recombinant Immunotoxin LMB-2 and Chemotherapy to Block Immunogenicity. *Clin. cancer Res. an Off. J. Am. Assoc. Cancer Res.* 22, 310–318. doi:10.1158/1078-0432.CCR-15-1412.

Kuan, C.-T., Wakiya, K., Keir, S. T., Li, J., Herndon, J. E. 2nd, Pastan, I., et al. (2011). Affinity-matured anti-glycoprotein NMB recombinant immunotoxins targeting malignant gliomas and melanomas. *Int. J. cancer* 129, 111–121. doi:10.1002/ijc.25645.

Kuan, C. T., and Pastan, I. (1996). Improved antitumor activity of a recombinant anti-Lewis(y) immunotoxin not requiring proteolytic activation. *Proc. Natl. Acad. Sci. U. S. A.* 93, 974–978. doi:10.1073/pnas.93.3.974.

Kunwar, S., Chang, S., Westphal, M., Vogelbaum, M., Sampson, J., Barnett, G., et al. (2010). Phase III randomized trial of CED of IL13-PE38QQR vs Gliadel wafers for recurrent glioblastoma. *Neuro. Oncol.* 12, 871–881. doi:10.1093/neuonc/nop054.

Langari, J., Karimipoor, M., Golkar, M., Khanahmad, H., Zeinali, S., Omidinia, S., et al. (2017). In Vitro Evaluation of Vegf-Pseudomonas Exotoxin: A Conjugated on Tumor Cells. *Adv. Biomed. Res.* 6, 144. doi:10.4103/2277-9175.218691.

Lee, B. S., Lee, Y., Park, J., Jeong, B. S., Jo, M., Jung, S. T., et al. (2019). Construction of an immunotoxin via site-specific conjugation of anti-Her2 IgG and engineered Pseudomonas exotoxin A. *J. Biol. Eng.* 13, 56. doi:10.1186/s13036-019-0188-x.

Lim, D., Kim, K. S., Kim, H., Ko, K.-C., Song, J. J., Choi, J. H., et al. (2017). Anti-tumor activity of an immunotoxin (TGFα-PE38) delivered by attenuated Salmonella typhimurium. *Oncotarget* 8, 37550–37560. doi:10.18632/oncotarget.17197.

Liu, Z., Feng, Z., Zhu, X., Xu, W., Zhu, J., Zhang, X., et al. (2013). Construction, expression, and characterization of an anti-tumor immunotoxin containing the human anti-c-Met single-chain antibody and PE38KDEL. *Immunol. Lett.* 149, 30–40. doi:10.1016/j.imlet.2012.09.006.

Lorberboum-Galski, H., Barrett, L. V, Kirkman, R. L., Ogata, M., Willingham, M. C., FitzGerald, D. J., et al. (1989). Cardiac allograft survival in mice treated with IL-2-PE40. *Proc. Natl. Acad. Sci. U. S. A.* 86, 1008–1012. doi:10.1073/pnas.86.3.1008.

Mareeva, T., Wanjalla, C., Schnell, M. J., and Sykulev, Y. (2010). A novel composite immunotoxin that suppresses rabies virus production by the infected cells. *J. Immunol. Methods* 353, 78–86. doi:10.1016/j.jim.2009.11.010.

Matsushita, S., Koito, A., Maeda, Y., Hattori, T., and Takatsuki, K. (1990). Selective killing of HIV-infected cells by anti-gp120 immunotoxins. *AIDS Res. Hum. Retroviruses* 6, 193–203. doi:10.1089/aid.1990.6.193.

Mazor, Y., Barnea, I., Keydar, I., and Benhar, I. (2007). Antibody internalization studied using a novel IgG binding toxin fusion. *J. Immunol. Methods* 321, 41–59. doi:10.1016/j.jim.2007.01.008.

Merriam, D., Chen, C., Mendez-Lagares, G., Rogers, K. A., Michaels, A. J., Yan, J., et al. (2017). Depletion of Gut-Resident CCR5(+) Cells for HIV Cure Strategies. *AIDS Res. Hum. Retroviruses* 33, S70–S80. doi:10.1089/aid.2017.0159.

Mickisch, G. H., Pai, L. H., Gottesman, M. M., and Pastan, I. (1992). Monoclonal antibody MRK16 reverses the multidrug resistance of multidrug-resistant transgenic mice. *Cancer Res.* 52, 4427–4432.

Mirzaee, M., Jalali-Javaran, M., Moieni, A., Zeinali, S., and Behdani, M. (2018). Expression of VGRNb-PE immunotoxin in transplastomic lettuce (Lactuca sativa L.). *Plant Mol. Biol.* 97, 103–112. doi:10.1007/s11103-018-0726-9.

Mladenov, R., Hristodorov, D., Cremer, C., Hein, L., Kreutzer, F., Stroisch, T., et al. (2015). The Fc-alpha receptor is a new target antigen for immunotherapy of myeloid leukemia. *Int. J. cancer* 137, 2729–2738. doi:10.1002/ijc.29628.

Nachreiner, T., Kampmeier, F., Thepen, T., Fischer, R., Barth, S., and Stocker, M. (2008). Depletion of autoreactive B-lymphocytes by a recombinant myelin oligodendrocyte glycoprotein-based immunotoxin. *J. Neuroimmunol.* 195, 28–35. doi:10.1016/j.jneuroim.2008.01.001.

Nagai, T., Kyo, A., Hasui, K., Takao, S., and Matsuyama, T. (2012). Efficacy of an immunotoxin to folate receptor beta in the intra-articular treatment of antigen-induced arthritis. *Arthritis Res. Ther.* 14, R106. doi:10.1186/ar3831.

Nagata, S., Onda, M., Numata, Y., Santora, K., Beers, R., Kreitman, R. J., et al. (2002). Novel anti-CD30 recombinant immunotoxins containing disulfide-stabilized Fv fragments. *Clin. Cancer Res.* 8, 2345–2355.

Nand, K. N., Gupta, J. C., Panda, A. K., and Jain, S. K. (2015). Development of a recombinant hCG-specific single chain immunotoxin cytotoxic to hCG expressing cancer cells. *Protein Expr. Purif.* 106, 10–17. doi:10.1016/j.pep.2014.10.008.

Nechushtan, A., Yarkoni, S., Marianovsky, I., and Lorberboum-Galski, H. (1997). Adenocarcinoma cells are targeted by the new GnRH-PE66 chimeric toxin through specific gonadotropin-releasing hormone binding sites. *J. Biol. Chem.* 272, 11597–11603. doi:10.1074/jbc.272.17.11597.

Nicholls, P. J., Johnson, V. G., Andrew, S. M., Hoogenboom, H. R., Raus, J. C., and Youle, R. J. (1993). Characterization of single-chain antibody (sFv)-toxin fusion proteins produced in vitro in rabbit reticulocyte lysate. *J. Biol. Chem.* 268, 5302–5308.

Niv, R., Assaraf, Y. G., Segal, D., Pirak, E., and Reiter, Y. (2001). Targeting multidrug resistant tumor cells with a recombinant single-chain FV fragment directed to P-glycoprotein. *Int. J. cancer* 94, 864–872. doi:10.1002/ijc.1552.

O’Brien, P., Smythe, A., Biggs, J. C., and Smith, G. M. (1997). A recombinant GM-CSF-PE40 ligand toxin is functionally active but not cytotoxic to cells. *Immunol. Cell Biol.* 75, 289–294. doi:10.1038/icb.1997.44.

Oh, S., Todhunter, D. A., Panoskaltsis-Mortari, A., Buchsbaum, D. J., Toma, S., and Vallera, D. A. (2012). A deimmunized bispecific ligand-directed toxin that shows an impressive anti-pancreatic cancer effect in a systemic nude mouse orthotopic model. *Pancreas* 41, 789–796. doi:10.1097/MPA.0b013e31823b5f2e.

Oh, S., Tsai, A. K., Ohlfest, J. R., Panoskaltsis-Mortari, A., and Vallera, D. A. (2011). Evaluation of a bispecific biological drug designed to simultaneously target glioblastoma and its neovasculature in the brain. *J. Neurosurg.* 114, 1662–1671. doi:10.3171/2010.11.JNS101214.

Onda, M., Kobayashi, K., and Pastan, I. (2019). Depletion of regulatory T cells in tumors with an anti-CD25 immunotoxin induces CD8 T cell-mediated systemic antitumor immunity. *Proc. Natl. Acad. Sci. U. S. A.* 116, 4575–4582. doi:10.1073/pnas.1820388116.

Onda, M., Olafsen, T., Tsutsumi, Y., Bruland, O. S., and Pastan, I. (2001). Cytotoxicity of antiosteosarcoma recombinant immunotoxins composed of TP-3 Fv fragments and a truncated Pseudomonas exotoxin A. *J. Immunother.* 24, 144–150.

Onda, M., Wang, Q., Guo, H., Cheung, N.-K. V, and Pastan, I. (2004). In Vitro and in Vivo Cytotoxic Activities of Recombinant Immunotoxin 8H9(Fv)-PE38 against Breast Cancer, Osteosarcoma, and Neuroblastoma. *Cancer Res.* 64, 1419 LP – 1424. doi:10.1158/0008-5472.CAN-03-0570.

Pai-Scherf, L. H., Villa, J., Pearson, D., Watson, T., Liu, E., Willingham, M. C., et al. (1999). Hepatotoxicity in cancer patients receiving erb-38, a recombinant immunotoxin that targets the erbB2 receptor. *Clin. Cancer Res.* 5, 2311–2315.

Pai, L. H., Bookman, M. A., Ozols, R. F., Young, R. C., Smith, J. W. 2nd, Longo, D. L., et al. (1991a). Clinical evaluation of intraperitoneal Pseudomonas exotoxin immunoconjugate OVB3-PE in patients with ovarian cancer. *J. Clin. Oncol.* 9, 2095–2103. doi:10.1200/JCO.1991.9.12.2095.

Pai, L. H., Gallo, M. G., FitzGerald, D. J., and Pastan, I. (1991b). Antitumor activity of a transforming growth factor alpha-Pseudomonas exotoxin fusion protein (TGF-alpha-PE40). *Cancer Res.* 51, 2808–2812.

Pai, L. H., Wittes, R., Setser, A., Willingham, M. C., and Pastan, I. (1996). Treatment of advanced solid tumors with immunotoxin LMB-1: an antibody linked to Pseudomonas exotoxin. *Nat. Med.* 2, 350–353. doi:10.1038/nm0396-350.

Peipp, M., Kupers, H., Saul, D., Schlierf, B., Greil, J., Zunino, S. J., et al. (2002). A recombinant CD7-specific single-chain immunotoxin is a potent inducer of apoptosis in acute leukemic T cells. *Cancer Res.* 62, 2848–2855.

Peng, Q.-S., Li, Y.-H., and Zhu, P. (2006). [High level expression, purification and cytotoxicity of IL-10(18-57)-PE40]. *Sheng Wu Gong Cheng Xue Bao* 22, 87–93.

Pichinuk, E., Chalik, M., Benhar, I., Ginat-Koton, R., Ziv, R., Smorodinsky, N. I., et al. (2020). In vivo anti-MUC1(+) tumor activity and sequences of high-affinity anti-MUC1-SEA antibodies. *Cancer Immunol. Immunother.* doi:10.1007/s00262-020-02547-2.

Pirker, R., FitzGerald, D. J., Hamilton, T. C., Ozols, R. F., Willingham, M. C., and Pastan, I. (1985). Anti-transferrin receptor antibody linked to Pseudomonas exotoxin as a model immunotoxin in human ovarian carcinoma cell lines. *Cancer Res.* 45, 751–757.

Posey, J. A., Khazaeli, M. B., Bookman, M. A., Nowrouzi, A., Grizzle, W. E., Thornton, J., et al. (2002). A phase I trial of the single-chain immunotoxin SGN-10 (BR96 sFv-PE40) in patients with advanced solid tumors. *Clin. Cancer Res.* 8, 3092–3099.

Proby, C. M., Ota, T., Suzuki, H., Koyasu, S., Gamou, S., Shimizu, N., et al. (2000). Development of chimeric molecules for recognition and targeting of antigen-specific B cells in pemphigus vulgaris. *Br. J. Dermatol.* 142, 321–330. doi:10.1046/j.1365-2133.2000.03328.x.

Rajagopal, V., and Kreitman, R. J. (2000). Recombinant toxins that bind to the urokinase receptor are cytotoxic without requiring binding to the alpha(2)-macroglobulin receptor. *J. Biol. Chem.* 275, 7566–7573. doi:10.1074/jbc.275.11.7566.

Ramachandran, R. V, Katzenstein, D. A., Wood, R., Batts, D. H., and Merigan, T. C. (1994). Failure of short-term CD4-PE40 infusions to reduce virus load in human immunodeficiency virus-infected persons. *J. Infect. Dis.* 170, 1009–1013. doi:10.1093/infdis/170.4.1009.

Rezaie, E., Amani, J., Bidmeshki Pour, A., and Mahmoodzadeh Hosseini, H. (2020). A new scfv-based recombinant immunotoxin against EPHA2-overexpressing breast cancer cells; High in vitro anti-cancer potency. *Eur. J. Pharmacol.* 870, 172912. doi:10.1016/j.ejphar.2020.172912.

Ribbert, T., Thepen, T., Tur, M. K., Fischer, R., Huhn, M., and Barth, S. (2010). Recombinant, ETA’-based CD64 immunotoxins: improved efficacy by increased valency, both in vitro and in vivo in a chronic cutaneous inflammation model in human CD64 transgenic mice. *Br. J. Dermatol.* 163, 279–286. doi:10.1111/j.1365-2133.2010.09824.x.

Roffler, S. R., Yu, M. H., Chen, B. M., Tung, E., and Yeh, M. Y. (1991). Therapy of human cervical carcinoma with monoclonal antibody-Pseudomonas exotoxin conjugates. *Cancer Res.* 51, 4001–4007.

Rozemuller, H., Chowdhury, P. S., Pastan, I., and Kreitman, R. J. (2001). Isolation of new anti-CD30 scFvs from DNA-immunized mice by phage display and biologic activity of recombinant immunotoxins produced by fusion with truncated pseudomonas exotoxin. *Int. J. cancer* 92, 861–870. doi:10.1002/ijc.1266.

Schmidt, M., and Wels, W. (1996). Targeted inhibition of tumour cell growth by a bispecific single-chain toxin containing an antibody domain and TGF alpha. *Br. J. Cancer* 74, 853–862. doi:10.1038/bjc.1996.448.

Schwemmlein, M., Peipp, M., Barbin, K., Saul, D., Stockmeyer, B., Repp, R., et al. (2006). A CD33-specific single-chain immunotoxin mediates potent apoptosis of cultured human myeloid leukaemia cells. *Br. J. Haematol.* 133, 141–151. doi:10.1111/j.1365-2141.2005.05869.x.

Schwemmlein, M., Stieglmaier, J., Kellner, C., Peipp, M., Saul, D., Oduncu, F., et al. (2007). A CD19-specific single-chain immunotoxin mediates potent apoptosis of B-lineage leukemic cells. *Leukemia* 21, 1405–1412. doi:10.1038/sj.leu.2404687.

Schwenkert, M., Birkholz, K., Schwemmlein, M., Kellner, C., Kugler, M., Peipp, M., et al. (2008). A single chain immunotoxin, targeting the melanoma-associated chondroitin sulfate proteoglycan, is a potent inducer of apoptosis in cultured human melanoma cells. *Melanoma Res.* 18, 73–84. doi:10.1097/CMR.0b013e3282f7c8f9.

Shancer, Z., Liu, X.-F., Nagata, S., Zhou, Q., Bera, T. K., and Pastan, I. (2019). Anti-BCMA immunotoxins produce durable complete remissions in two mouse myeloma models. *Proc. Natl. Acad. Sci. U. S. A.* 116, 4592–4598. doi:10.1073/pnas.1821733116.

Shapira, S., Shapira, A., Starr, A., Kazanov, D., Kraus, S., Benhar, I., et al. (2011). An immunoconjugate of anti-CD24 and Pseudomonas exotoxin selectively kills human colorectal tumors in mice. *Gastroenterology* 140, 935–946. doi:10.1053/j.gastro.2010.12.004.

Siegall, C. B., Bacus, S. S., Cohen, B. D., Plowman, G. D., Mixan, B., Chace, D., et al. (1995). HER4 expression correlates with cytotoxicity directed by a heregulin- toxin fusion protein. *J. Biol. Chem.* 270, 7625–7630. doi:10.1074/jbc.270.13.7625.

Siegall, C. B., Kreitman, R. J., FitzGerald, D. J., and Pastan, I. (1991). Antitumor effects of interleukin 6-Pseudomonas exotoxin chimeric molecules against the human hepatocellular carcinoma, PLC/PRF/5 in mice. *Cancer Res.* 51, 2831–2836.

Siegall, C. B., Nordan, R. P., FitzGerald, D. J., and Pastan, I. (1990a). Cell-specific toxicity of a chimeric protein composed of interleukin-6 and Pseudomonas exotoxin (IL6-PE40) on tumor cells. *Mol. Cell. Biol.* 10, 2443–2447. doi:10.1128/mcb.10.6.2443-2447.1990.

Siegall, C. B., Schwab, G., Nordan, R. P., FitzGerald, D. J., and Pastan, I. (1990b). Expression of the interleukin 6 receptor and interleukin 6 in prostate carcinoma cells. *Cancer Res.* 50, 7786–7788.

Simon, N., Antignani, A., Sarnovsky, R., Hewitt, S. M., and FitzGerald, D. (2016). Targeting a Cancer-Specific Epitope of the Epidermal Growth Factor Receptor in Triple-Negative Breast Cancer. *J. Natl. Cancer Inst.* 108. doi:10.1093/jnci/djw028.

Singh, R., Samant, U., Hyland, S., Chaudhari, P. R., Wels, W. S., and Bandyopadhyay, D. (2007). Target-specific cytotoxic activity of recombinant immunotoxin scFv(MUC1)-ETA on breast carcinoma cells and primary breast tumors. *Mol. Cancer Ther.* 6, 562–569. doi:10.1158/1535-7163.MCT-06-0604.

Sokolova, E. A., Shilova, O. N., Kiseleva, D. V, Schulga, A. A., Balalaeva, I. V, and Deyev, S. M. (2019). HER2-Specific Targeted Toxin DARPin-LoPE: Immunogenicity and Antitumor Effect on Intraperitoneal Ovarian Cancer Xenograft Model. *Int. J. Mol. Sci.* 20. doi:10.3390/ijms20102399.

Sokolova, E. A., Stremovskiy, O. A., Zdobnova, T. A., Balalaeva, I. V, and Deyev, S. M. (2015). Recombinant Immunotoxin 4D5scFv-PE40 for Targeted Therapy of HER2-Positive Tumors. *Acta Naturae* 7, 93–96.

Sokolova, E., Proshkina, G., Kutova, O., Shilova, O., Ryabova, A., Schulga, A., et al. (2016). Recombinant targeted toxin based on HER2-specific DARPin possesses a strong selective cytotoxic effect in vitro and a potent antitumor activity in vivo. *J. Control. Release* 233, 48–56. doi:10.1016/j.jconrel.2016.05.020.

Song, J., Ren, H., Li, Y., Xu, J., Kong, H., Tong, W., et al. (2013). rG17PE38, a novel immunotoxin target to gastric cancer with overexpressed CCK-2R. *J. Drug Target.* 21, 375–382. doi:10.3109/1061186X.2012.757770.

Staudinger, M., Glorius, P., Burger, R., Kellner, C., Klausz, K., Gunther, A., et al. (2014). The novel immunotoxin HM1.24-ETA’ induces apoptosis in multiple myeloma cells. *Blood Cancer J.* 4, e219. doi:10.1038/bcj.2014.38.

Stein, C., Kellner, C., Kugler, M., Reiff, N., Mentz, K., Schwenkert, M., et al. (2010). Novel conjugates of single-chain Fv antibody fragments specific for stem cell antigen CD123 mediate potent death of acute myeloid leukaemia cells. *Br. J. Haematol.* 148, 879–889. doi:10.1111/j.1365-2141.2009.08033.x.

Tang, J., Li, J., Zhu, X., Yu, Y., Chen, D., Yuan, L., et al. (2016). Novel CD7-specific nanobody-based immunotoxins potently enhanced apoptosis of CD7-positive malignant cells. *Oncotarget* 7, 34070–34083. doi:10.18632/oncotarget.8710.

Terrier, B., Nagata, S., Ise, T., Rosenzwajg, M., Pastan, I., Klatzmann, D., et al. (2014). CD21(-/low) marginal zone B cells highly express Fc receptor-like 5 protein and are killed by anti-Fc receptor-like 5 immunotoxins in hepatitis C virus-associated mixed cryoglobulinemia vasculitis. *Arthritis Rheumatol. (Hoboken, N.J.)* 66, 433–443. doi:10.1002/art.38222.

Theuer, C. P., Fitzgerald, D. J., and Pastan, I. (1993a). A recombinant form of Pseudomonas exotoxin A containing transforming growth factor alpha near its carboxyl terminus for the treatment of bladder cancer. *J. Urol.* 149, 1626–1632. doi:10.1016/S0022-5347(17)36464-9.

Theuer, C. P., FitzGerald, D., and Pastan, I. (1992). A recombinant form of Pseudomonas exotoxin directed at the epidermal growth factor receptor that is cytotoxic without requiring proteolytic processing. *J. Biol. Chem.* 267, 16872–16877. doi:https://doi.org/10.1016/S0021-9258(18)41865-0.

Theuer, C. P., Kreitman, R. J., FitzGerald, D. J., and Pastan, I. (1993b). Immunotoxins made with a recombinant form of Pseudomonas exotoxin A that do not require proteolysis for activity. *Cancer Res.* 53, 340–347.

Trad, A., Hansen, H. P., Shomali, M., Peipp, M., Klausz, K., Hedemann, N., et al. (2013). ADAM17-overexpressing breast cancer cells selectively targeted by antibody-toxin conjugates. *Cancer Immunol. Immunother.* 62, 411–421. doi:10.1007/s00262-012-1346-x.

Tur, M. K., Sasse, S., Stocker, M., Djabelkhir, K., Huhn, M., Matthey, B., et al. (2001). An anti-GD2 single chain Fv selected by phage display and fused to Pseudomonas exotoxin A develops specific cytotoxic activity against neuroblastoma derived cell lines. *Int. J. Mol. Med.* 8, 579–584. doi:10.3892/ijmm.8.5.579.

Vallera, D. A., Jin, N., Shu, Y., Panoskaltsis-Mortari, A., Kelekar, A., and Chen, W. (2003). Retroviral immunotoxin gene therapy of leukemia in mice using leukemia-specific T cells transduced with an interleukin-3/Bax fusion protein gene. *Hum. Gene Ther.* 14, 1787–1798. doi:10.1089/104303403322611791.

Waldron, N. N., Kaufman, D. S., Oh, S., Inde, Z., Hexum, M. K., Ohlfest, J. R., et al. (2011). Targeting tumor-initiating cancer cells with dCD133KDEL shows impressive tumor reductions in a xenotransplant model of human head and neck cancer. *Mol. Cancer Ther.* 10, 1829–1838. doi:10.1158/1535-7163.MCT-11-0206.

Wang, C., Gao, W., Feng, M., Pastan, I., and Ho, M. (2017). Construction of an immunotoxin, HN3-mPE24, targeting glypican-3 for liver cancer therapy. *Oncotarget* 8, 32450–32460. doi:10.18632/oncotarget.10592.

Wang, D., Deng, X., Leng, X., and Mao, X. (2010). Interleukin-15 receptor-directed immunotoxins atteunuate disease severity in rat adjuvant arthritis. *Mol. Immunol.* 47, 1535–1543. doi:10.1016/j.molimm.2010.01.023.

Wang, H., Dai, J., Li, B., Fan, K., Peng, L., Zhang, D., et al. (2008). Expression, purification, and characterization of an immunotoxin containing a humanized anti-CD25 single-chain fragment variable antibody fused to a modified truncated Pseudomonas exotoxin A. *Protein Expr. Purif.* 58, 140–147. doi:10.1016/j.pep.2007.09.009.

Wang, H., Song, S., Kou, G., Li, B., Zhang, D., Hou, S., et al. (2007). Treatment of hepatocellular carcinoma in a mouse xenograft model with an immunotoxin which is engineered to eliminate vascular leak syndrome. *Cancer Immunol. Immunother.* 56, 1775–1783. doi:10.1007/s00262-007-0321-4.

Wang, S., Li, L., Shi, R., Liu, X., Zhang, J., Zou, Z., et al. (2016). Mast Cell Targeted Chimeric Toxin Can Be Developed as an Adjunctive Therapy in Colon Cancer Treatment. *Toxins (Basel).* 8. doi:10.3390/toxins8030071.

Weber, F., Asher, A., Bucholz, R., Berger, M., Prados, M., Chang, S., et al. (2003). Safety, tolerability, and tumor response of IL4-Pseudomonas exotoxin (NBI-3001) in patients with recurrent malignant glioma. *J. Neurooncol.* 64, 125–137. doi:10.1007/BF02700027.

Wolf, P., Alt, K., Buhler, P., Katzenwadel, A., Wetterauer, U., Tacke, M., et al. (2008). Anti-PSMA immunotoxin as novel treatment for prostate cancer? High and specific antitumor activity on human prostate xenograft tumors in SCID mice. *Prostate* 68, 129–138. doi:10.1002/pros.20684.

Wolf, P., Alt, K., Wetterauer, D., Buhler, P., Gierschner, D., Katzenwadel, A., et al. (2010). Preclinical evaluation of a recombinant anti-prostate specific membrane antigen single-chain immunotoxin against prostate cancer. *J. Immunother.* 33, 262–271. doi:10.1097/CJI.0b013e3181c5495c.

Wolff, E. A., Hellstrom, I., Chace, D. F., Hellstrom, K. E., and Siegall, C. B. (1995). Antitumour activity of a melanoma-specific immunotoxin, ME20-LysPE40. *Ther. Immunol.* 2, 137–145.

Xi, Y., Yuan, Z., Zhang, H., Guan, H., Kong, F., Liu, N., et al. (2006). Molecular construction and characterization of a novel exotoxin fusion protein that selectively blocks the B7:CD28 costimulatory signal system. *J. Immunother.* 29, 586–595. doi:10.1097/01.cji.0000211300.67750.71.

Xiong, W., Huang, N., Feng, Y., Wu, Q., and Wang, B. (2008). Creation and anti-cancer potency in HeLa cells of a novel chimeric toxin, HMGNCIDIN, composed of HMGN2 a-helical domain and PE38 KDEL domain III. *Chin. Med. J. (Engl).* 121, 82–85.

Xu, J., Li, X., Jiang, B., Feng, X., Wu, J., Cai, Y., et al. (2018). Antiviral Immunotoxin Against Bovine herpesvirus-1: Targeted Inhibition of Viral Replication and Apoptosis of Infected Cell. *Front. Microbiol.* 9, 653. doi:10.3389/fmicb.2018.00653.

Yang, D., Kuan, C. T., Payne, J., Kihara, A., Murray, A., Wang, L. M., et al. (1998). Recombinant heregulin-Pseudomonas exotoxin fusion proteins: interactions with the heregulin receptors and antitumor activity in vivo. *Clin. Cancer Res.* 4, 993–1004.

Yang, H., He, D., Chao, K., Lin, Q., You, S., and Huang, H.-L. (2004). [Studies of the expression, purification, renaturation and biologic activity of an anti-CEA immunotoxin]. *Sheng Wu Gong Cheng Xue Bao* 20, 348–351.

Yao, Q., Zheng, Q.-M., Wen, J.-F., Lu, T., Wei, M.-Q., and Dai, S.-Z. (2010). [Target-specific cytotoxic activity of recombinant fusion toxin C-CPE-ETA’ against CLDN-3,4-overexpressing ovarian cancer cells]. *Zhonghua Zhong Liu Za Zhi* 32, 897–902.

Yu, Y., Li, J., Zhu, X., Tang, X., Bao, Y., Sun, X., et al. (2017). Humanized CD7 nanobody-based immunotoxins exhibit promising anti-T-cell acute lymphoblastic leukemia potential. *Int. J. Nanomedicine* 12, 1969–1983. doi:10.2147/IJN.S127575.

Zangemeister-Wittke, U., Collinson, A. R., Froschl, B., Waibel, R., Schenker, T., and Stahel, R. A. (1994). Immunotoxins recognising a new epitope on the neural cell adhesion molecule have potent cytotoxic effects against small cell lung cancer. *Br. J. Cancer* 69, 32–39. doi:10.1038/bjc.1994.5.

Zdobnova, T., Sokolova, E., Stremovskiy, O., Karpenko, D., Telford, W., Turchin, I., et al. (2015). A novel far-red fluorescent xenograft model of ovarian carcinoma for preclinical evaluation of HER2-targeted immunotoxins. *Oncotarget* 6, 30919–30928. doi:10.18632/oncotarget.5130.

Zhao, P., Wang, P., Dong, S., Zhou, Z., Cao, Y., Yagita, H., et al. (2019). Depletion of PD-1-positive cells ameliorates autoimmune disease. *Nat. Biomed. Eng.* 3, 292–305. doi:10.1038/s41551-019-0360-0.

Zhu, S., Chen, J., Xiong, Y., Kamara, S., Gu, M., Tang, W., et al. (2020). Novel EBV LMP-2-affibody and affitoxin in molecular imaging and targeted therapy of nasopharyngeal carcinoma. *PLoS Pathog.* 16, e1008223. doi:10.1371/journal.ppat.1008223.

Zhu, X., Tao, K., Li, Y., Li, S., Zhang, L., Wang, D., et al. (2013). A new recombinant immunotoxin hscFv-ETA’ demonstrates specific cytotoxicity against chronic myeloid leukemia cells in vitro. *Immunol. Lett.* 154, 18–24. doi:10.1016/j.imlet.2013.08.002.

Zielinski, R., Lyakhov, I., Jacobs, A., Chertov, O., Kramer-Marek, G., Francella, N., et al. (2009). Affitoxin--a novel recombinant, HER2-specific, anticancer agent for targeted therapy of HER2-positive tumors. *J. Immunother.* 32, 817–825. doi:10.1097/CJI.0b013e3181ad4d5d.
